# Activation of basolateral amygdala to anterior cingulate cortex circuit alleviates MK-801 induced social and cognitive deficits of schizophrenia

**DOI:** 10.3389/fncel.2022.1070015

**Published:** 2022-12-22

**Authors:** Xin Huang, Yaohao Li, Haiying Liu, Jinwei Xu, Zehua Tan, Haoyang Dong, Biqing Tian, Shengxi Wu, Wenting Wang

**Affiliations:** Department of Neurobiology, School of Basic Medicine, Fourth Military Medical University, Xi’an, Shaanxi, China

**Keywords:** MK-801, schizophrenia, anterior cingulate cortex (ACC), basolateral amygdala (BLA), cognitive deficits, social deficits, stereotypic rotational behavior

## Abstract

**Introduction:**

Schizophrenia is a severe psychiatric disorder with a high prevalence worldwide, however, its pathogenesis remains poorly understood.

**Methods and results:**

In this study, we used the non-competitive NMDA receptor antagonist MK-801 to induce schizophrenia-like behaviors and confirmed that mice exhibited stereotypic rotational behavior and hyperlocomotion, social interaction defects and cognitive dysfunction, similar to the clinical symptoms in patients. Here, the anterior cingulate cortex (ACC) and basolateral amygdala (BLA) were involved in the schizophrenia-like behaviors induced by MK-801. Furthermore, we confirmed BLA sent glutamatergic projection to the ACC. Chemogenetic and optogenetic regulation of BLA-ACC projecting neurons affected social and cognitive deficits but not stereotypic rotational behavior in MK-801-treated mice.

**Discussion:**

Overall, our study revealed that the BLA-ACC circuit plays a major role and may be a potential target for treating schizophrenia-related symptoms.

## Introduction

Schizophrenia is a chronic, severe psychiatric disorder with a global prevalence of approximately 1% (Lauriello, 2020). Clinically, it is characterized by three types of core symptoms: positive symptoms (hallucinations, paranoia, and thought disorders), negative symptoms (social withdrawal, lack of motivation, and poor attention), and cognitive deficits (impaired executive function, working memory, and attention) (Winship et al., 2019). Because of these three types of symptoms, schizophrenia exerts a profound effect on the patient’s life and imposes a huge burden on the health care system and society as a whole. However, the etiology and pathogenesis of schizophrenia have not been sufficiently clarified.

In recent years, researchers have shown an increased interest in the abnormalities of glutamatergic neurotransmission in individuals with schizophrenia (Howes et al., 2015). The reduction in N-methyl-D-aspartate (NMDA) receptor expression and pharmacological inhibition of NMDA receptors were found to reproduce all three core symptoms associated with the disease (Grotta et al., 1995). Hence, non-competitive NMDA receptor antagonists, such as MK-801 (also known as dizocilpine), have been frequently administered to generate animal models of schizophrenia (Gallant et al., 2017). Animals exposed to MK-801 exhibit behaviors and neurochemical alterations similar to those observed in patients with schizophrenia (Murueta-Goyena et al., 2019). In rodents, systemic administration of NMDA receptor antagonists caused increased locomotor activity and stereotypic behaviors, which are commonly used as an indication of positive symptoms of schizophrenia. Changes in social interaction are used as an indicator of negative symptoms. NMDA receptor antagonists also disrupt different domains of cognition (Jones et al., 2011). Using animal models, some new therapeutics have been developed based on the understanding of the mechanisms underlying disease pathology in patients with schizophrenia (Buck et al., 2022).

Despite the aforementioned discoveries, the understanding of the pathology of schizophrenia is far from sufficient. The pathogenesis of schizophrenia is notably very complex. It is characterized by multiple clinical symptoms, indicating that far more than one brain region is involved. Previous studies have shown that surgical ablations of certain corticolimbic regions, such as the anterior cingulate cortex (ACC), hippocampal formation (HIP), and basolateral amygdala (BLA) (Rolls, 2019), result in changes in several features of personality, which is abnormal in individuals with schizophrenia (Prestia et al., 2015; Bryll et al., 2020). The ACC is a key region of the limbic system that mediates a wide range of functions, such as the regulation of emotion, action and memory, executive control, and social behavior (Rolls, 2019). Because perturbations of many aspects of these functions are commonly observed in patients with schizophrenia, converging lines of evidence from postmortem and neuroimaging studies have consistently revealed that the ACC is structurally and functionally altered in individuals with schizophrenia (Benes, 1998). The BLA is part of a frontotemporal system that innervates several key components of the corticolimbic system, including the ACC and hippocampal formation (Janak and Tye, 2015). Animals with extensive amygdala ablations seem to lose the fear response completely; interestingly, these changes are very similar to those observed in patients with schizophrenia (Das et al., 2007). However, the abnormalities of specific mechanisms related to neural circuitry, which is defined by their afferent and efferent connectivity within key corticolimbic regions, are poorly understood in patients with schizophrenia (Benes, 2010).

In the current study, we used a mouse model of MK-801-induced schizophrenia to determine the roles of the ACC and BLA in schizophrenia-like behaviors. Furthermore, we evaluated the effect of modulating the neural circuitry on these behaviors to understand its etiology and increase our knowledge of the brain pathology in individuals with schizophrenia.

## Materials and methods

### Animals

We obtained adult C57BL/6J mice from the Fourth Military Medical University (FMMU) animal facility. Glutamic acid decarboxylase-green fluorescence protein (GAD67 GFP) mice were a kind gift from Dr. Nobuaki Tamamaki, Kumamoto University (Tamamaki et al., 2003). GCaMP6s mice were purchased from Jackson Laboratory (Stock no. 024275). All animals used in this study were 6- to 8-week-old male mice and weighed approximately 20–25 g. They were housed in groups (4–6 mice per cage) on a 12/12-h light/dark cycle at a constant temperature (24°C) and had access to food and water *ad libitum*. All experimental procedures were approved by the Institutional Animal Care and Use Committee (IACUC-20200356) of the Fourth Military Medical University and were performed according to the “Principles of Medical Laboratory Animal Care” issued by the National Ministry of Health in China. All efforts were made to minimize animal suffering and to reduce the animals number used in this study.

### Experimental design

Adult male C57BL/6 mice (6–8 weeks) were obtained from the Experimental Animal Center of Fourth Military Medical University (Xi’an, China) and then were weighed and randomly assigned to the control group or the experimental group at a 1:1 ratio. The mice were acclimated for at least 7 days in the holding room before the experimental test, and they were gently handled for 3–5 min each day by the experimenters for seven consecutive days before behavior tests. For optogenetic manipulations and fiber photometry recording, mice were connected to the patch cable and then allowed to recover for 1–3 min before all behavioral tests were initiated. Considering the possible effects of multiple behavioral tests, different groups were used to explore positive behavior, negative behavior, and cognitive behavior. After the end of the behavioral tests, the brain tissues were removed from the mice for immunofluorescence histochemistry, and the virus injection sites were confirmed, the mice with absence or mis-placed virus injection, incorrect fiber position or drop of ceramic cartridge were excluded from experiments.

### Drugs

(+)-MK-801-hydrogen-maleate (M107, Sigma-Aldrich, USA) was freshly dissolved in Dimethyl sulfoxide (DMSO) first, and then used sterilized normal saline to dilute to the different concentration for intraperitoneal injected. Based on the results of our preliminary experiment, 0.2 mg/kg was finally selected, and the behavioral recording was started 15 min after the injection. The control group of mice was injected with the same volume of vehicle in the same manner. For the Designer Receptor Exclusively Activated by Designer Drugs (DREADD) manipulation before behavioral experiments, DREADD agonist 21 dihydrochloride (#6422, Tocris, USA, C21) was freshly dissolved in 0.9% sterile saline and intraperitoneally administered 3 weeks after DREADD protein expression and 30 min before behavioral tests were performed at a dose of 2 mg/kg.

### Behavioral tests

Before the behavioral tests, the mice were gently transferred to the testing room and allowed to acclimate to the environment for at least 1 h. The lighting was ∼ 100 lux in the experimental room to reduce the stimulation of the mice. All apparatuses were cleaned with 75% ethanol at the end of each behavioral test to remove olfactory cues.

According to diagnostic and statistical manual of mental disorders 5th ed. (DSM-V) diagnostic criteria, the symptoms of schizophrenia are mainly divided into three categories: positive symptoms, negative symptoms, and cognitive impairment (Tandon et al., 2013). We comprehensively evaluated the changes in mouse behavior caused by MK-801 by assessing positive behaviors using open field test (OFT) and rotational behavior experiments and quantifying negative behaviors using the social interaction test. The novel object recognition (NOR) test was used to explore cognitive impairment, and a gait test was used to study whether MK-801 could cause other concomitant symptoms (such as dysfunction in locomotor coordination) in mice.

### Rotational behavior test

The rotational behavior of mice was measured in a small non-transparent plastic box (20 cm × 20 cm × 25 cm) after the MK-801 or vehicle injection. An infrared camera mounted on the top of the box was used to record behavior. During the concentration test and incubation period assessment, the behavioral recording started immediately after drug injection until the rotational behavior gradually decreased. Finally, according to the previous experimental results, we chose to conduct recordings 15 min after the drug injection, and each mouse was recorded for 10 min. The experimental device was cleaned thoroughly with 75% alcohol after each trial. The total rotating laps performed by the mouse were analyzed using SMART v.3.0 software (Panlab Harvard Apparatus, Spain) by the experimenters who were blinded to the groups.

### Open field test

The OFT was used to evaluate the locomotor ability of mice, as previously described (Liu et al., 2022). The OFT device was a light gray polyvinyl chloride box (50 cm × 50 cm × 50 cm). Each mouse was gently placed into the central zone of the box from their home cage, and an overhead camera connected to a computer tracked the movements of the mice. After 10 min of recording, we cleaned the entire area of the box with 75% ethanol before proceeding to recording the next test animal. The total distance traveled by the mice was analyzed by the experimenters who were blinded to the groups using SMART v.3.0 software.

### Social interaction test

A 10-min free social interaction test was performed to examine social behavior when subject animals freely interacted with a juvenile animal, as previously described (Guo et al., 2019). The test was conducted in the mouse’s home cage with an overhead camera connected to a computer to record the mouse’s behavior. An unfamiliar juvenile male mouse was introduced into the cage for free interaction (different juveniles were used in different tests). The total social time and social bouts were annotated and quantified by the experimenters who were blinded to the groups using SMART v.3.0 software.

### Novel object recognition test

The NOR test was performed as previously described with minor modifications (Sawahata et al., 2021). Mice were individually habituated to an OFT box for 3 days. During the training session, two novel objects composed of the same material but different colors and appearances were placed in the box, and mice were allowed to explore for 5 min to become familiar with them. An overhead camera recorded the behavior of the animals. In the test session, mice were gently placed back into the same box 24 h after the training session where one of the familiar objects was replaced by a novel object with the same material but a different color and appearance, and mice were allowed to explore freely for 5 min. The box was cleaned with 75% ethanol before proceeding to the next test. Experimenters who were blinded to the groups measured cognitive function by calculating the amount of time spent exploring the novel object in the test session and exploratory preference (the ratio of the amount of time spent exploring the novel object in the test session to the total time spent exploring both objects) using SMART v.3.0 software.

### CatWalk test

CatWalk XT (v10.6, Noldus Information Technology Inc., Netherlands) was used as previously reported to quantitatively assess gait function and motor performance as a complication of schizophrenia (Garrick et al., 2021). The apparatus consisted of a 1.3 m long transparent glass corridor with dim green light internally reflected into the glass walkway, with a high-speed video camera placed beneath the glass to record the walking patterns. The experiment was performed in a darkened room with red light and sheltered from noise. Each mouse was repeated three consecutive times, and average parameters were recorded. A trial was regarded as compliant if the mouse ran over the defined field of the camera (40 cm) without a reverse course and met the following criteria: run duration ranging from 3 to 30 s, run speed variation of 60%, and subsequent steps were more than seven steps. All gait characteristics were calculated with a special focus on (1) temporal parameters: stand time (time of paw contact with the glass), swing time (time the paw was not contacting the glass), step cycle (stand duration + swing duration); (2) spatial patterns: stride length (the distance between successive placements of the same paw), print length and width (the length/width of the complete print for that paw); and (3) speed- or velocity-related parameters: swing speed and body speed (Ungvari et al., 2017; Garrick et al., 2021).

### Virus injection and fiber implantation

For the virus injection, C57BL/6J mice were anesthetized with isoflurane, and their heads were fixed in a stereotaxic injection frame (#68016, RWD Life Science Inc., China). All viral injections were performed using a 10-μL microinjection needle (0.7 mm tip diameter, Shanghai Gaoge Industrial and Trade, China) to deliver the virus at a rate of 30 nL min^–1^ using a microsyringe pump (Legato 130, KD Scientific, USA). The needle was held at the site for 10 min to allow virus diffusion after injection. For the analysis of the projection of nerve fibers between two brain regions, 150 nL of rAAV-hSyn-EGFP-WPRE-pA (Cat#: PT-0905, titer: 2.01 × 10^12^ vg/ml, BrainVTA, China) was unilaterally injected into the BLA (−1.4 mm AP, +/−3.12 mm ML, −4.8 mm DV) of C57BL/6J mice, and 100 nL of rAAV2/retro-hSyn-Cre-mCherry-WPRE-hGH pA virus (Cat#: PT-0407, titer: 4.85 × 10^12^ vg/ml, BrainVTA, China) was unilaterally injected into the ACC (1.0 mm AP, +/−0.35 mm ML, −1.8 mm DV) of GAD67-GFP mice. For behavioral experiments, AAVs were bilaterally injected to the ACC or BLA. For the DREADD activation and subsequent behavioral tests, 150 nL of rAAV-EF1a-DIO-(Gq/GFP/Gi)-WPRE-bGHpA (Cat#: PT-0988/PT-0795/PT-0987, titer: 5.49/5.25/2.76 × 10^12^ vg/ml, BrainVTA, China) were bilaterally injected into the BLA, and 100 nL of AAV2/Retro-hSyn-NLS-Cre-P2A-mCherry were bilaterally injected into the ACC. For optical activation and behavioral experiments, 150 nL of rAAV-hSyn-(ChR2/mCherry/NpHR)-WPRE-pA (Cat#: PT-0002/PT-0013/PT-0007, titer: 4.05/5.25/5.10 × 10^12^ vg/ml, BrainVTA, China) were bilaterally injected into the BLA, and optical fibers (230 μm OD, NA 0.37) were implanted in the bilateral ACC (1.0 mm AP, +/−0.95 mm ML, −1.27 mm DV) at a 20 degrees-angle on the same day after the viral injection. The coordinates for the optic fiber were 200–300 mm above the viral injection coordinates. For fiber photometry recording, optical fibers (230 μm OD, NA0.37) were implanted immediately in the unilateral ACC (1.0 mm AP, −0.35 mm ML, −1.50 mm DV) and BLA (−1.4 mm AP, −3.12 mm ML, −4.5 mm DV) of the Gcamp6s mice. None of the mice underwent any behavioral tests for at least 3 weeks to allow them to recover from the surgeries. Data from only a *post hoc* analysis of viral targeting indicated that the injection coordinates were accurate.

### *In vivo* DREADD manipulation and behavioral tests

For the DREADD manipulation and behavioral experiments, C21 was freshly dissolved in sterile normal saline and intraperitoneally administered 3 weeks after the AAV2/8-EF1a-DIO-hM3D (Gq/GFP/Gi) injection and 30 min before behavioral tests were performed at a dose of 2 mg/kg based on our earlier experimental basis and previous reports (Jendryka et al., 2019; Goutaudier et al., 2020).

### *In vivo* optogenetic manipulations and behavioral tests

For optical stimulation in the behavioral tests, the patch cord was connected to the laser, and the fiber could freely rotate through a fiber-optic rotary connected with the patch cord. For the rotational behavior, OFT and NOR tests, photoactivation of ACC projecting fiber terminals from BLA was induced by applying 20-Hz light trains with 5-ms pulses of blue light generated by a 473-nm diode-pumped solid-state laser (QAXK-LASER-SS, Thinker Tech Nanjing Biotech Limited Co., China) or constant yellow light generated by a 594-nm diode-pumped solid-state laser (QAXK-LASER-YS, Thinker Tech Nanjing Biotech Limited Co., China) with the light power around 10 mW in the terminal of patch cord. For the social interaction test and OFT, the 10-min test was divided into two 5-min phases: laser stimulation off and on (OFF-ON phases). For the rotational behavior, the 30-min test was divided into three 10-min phases: laser stimulation off, on, and off (OFF-ON-OFF phases).

### Fiber photometry recording

Optical fibers were implanted into the ACC and BLA of Gcamp6s mice. An optical fiber guided the light between the commutator and the implanted optical fiber. The laser power at the tip of the optical fiber was adjusted to 0.01–0.02 mW to decrease laser bleaching. Fluorescence was bandpass-filtered (MF525-39, Thorlabs), and an amplifier was used to convert the photomultiplier tube current output to a voltage signal. The analog voltage signals were digitalized at 50 Hz and recorded by a fiber photometry system (Thinker Tech). Fiber photometry system (QAXK-MC-FP, ThinkerTech, Nanjing, China) is equipped with high-speed behavior industrial camera (Galaxy Series, Daheng imaging, China) system. This behavior camera can be controlled by software to output transistor-transistor logic (TTL) signal through general-purpose input/output (GPIO) ports synchronized with each frame and acquired by Data Acquisition Box (USB6001, National Instrument, USA), which also controlled the fiber photometry acquisition frequency. The data of fiber photometry is acquired by ThinkerTech MultiChannel FiberPhotometry GUI Software based on Labview (Thinker Tech Nanjing Biotech Limited Co., China). In a recording cycle, the number of behavior events divided by the time is the event frequency, which we defined as trail. In each trail, behavior onset has different definition, that is the moment mice touched the objects/mice in NOR test/social interaction test and the start of rotation (with a distinct body bend). To distinguish between rotation and running in rotational behavior test, we first analyzed behavior video in SMART v.3.0 software to get the onset time of rotation automatically, then we match it in the fiber photometry system.

The data were normalized (ΔF/F) by calculating the median signal across the recording period, subtracting this value from each data point, and dividing by the median signal. The area under the curve (AUCs) of ΔF/F in each defined time window were also calculated to quantify the change in fluorescence values induced by specific behavioral events. The heatmap and averaged Ca^2+^ traces were plotted using MATLAB program.

### Immunostaining and confocal imaging

After the behavioral experiments, the mice were anesthetized with isoflurane and transcardially perfused with 20 ml of 0.01 M phosphate buffered saline (pH 7.4), followed by 40 ml of 4% (w/v) paraformaldehyde (pH 7.4). For c-Fos staining, the mice were gently returned to their home cage and housed for 90 min without disturbing them before sacrificed. Brains were removed from the skull and then postfixed with 4% (w/v) paraformaldehyde at 4°C for 4 h. After cryoprotection with 30% (w/v) sucrose at 4°C for 48 h, the dehydrated brains were sectioned into 50 μm thick coronal slices using a freezing microtome (VT-1000, Leica Microsystems, Germany). Representative slices of the ACC and BLA were chosen and counterstained with Hoechst (ab228551, 1:1000, Abcam, USA) before imaging with a microscope (VS200, Olympus, Japan) to confirm the locations of the virus injection and implanted optical fibers. For c-Fos staining, the brain slices from each group were washed with 0.01 M PBS, rinsed with blocking solution at room temperature for 2 h, and incubated with primary antibodies against c-Fos (#2250S, 1:1500, Cell Signaling Technology, USA) overnight (4°C). The slices were washed with 0.01 M PBS the next day and probed with Alexa Fluor 488-conjugated anti-rabbit IgG (R37118, 1:500, Invitrogen, USA) secondary antibodies at room temperature for 4 h. The microscopic fields of these slices were imaged using a confocal microscope objective (VS200) and processed with Imaris software (v.9.5.0, Bitplane, Switzerland).

### *In situ* hybridization histochemistry

We performed *in situ* hybridization histochemistry as previously described (Guo et al., 2019). The standard protocol was that the mice were anesthetized with isoflurane and then perfused with 20 mL of 0.01 M diethylpyrocarbonate-treated phosphate-buffered saline (DEPC-PBS, pH 7.4) transcardially, followed by 40 mL of 4% (w/v) paraformaldehyde (pH 7.4). The brains were removed from the skull and postfixed with 4% (w/v) paraformaldehyde at 4°C for 4 h. All tubes and surgical equipment used were RNase-free. After cryoprotection in 30% (w/v) sucrose in 0.1 M DEPC–PB for 48 h at 4°C, the brains were cut into 50 μm thick coronal sections using a freezing microtome (VT-1000). Representative slices of ACC and BLA were chosen and washed with 0.01 M DEPC-PBS. The sections were incubated with 2% (v/v) H_2_O_2_ in 0.1 M DEPC-PB at room temperature for 10 min and then treated with 0.3% (v/v) Triton X-100 (# T8787, Millipore, USA) in 0.1 M DEPC–PBS at room temperature for 20 min. Next, the sections were acetylated in an acetylation solution for 10 min at room temperature. After two washes with DEPC–PBS, the sections were incubated with hybridization buffer for 1 h at 58°C. The sections were then incubated with 1 μg/ml *Vglut2* cRNA probe in hybridization buffer at 58°C for 18–20 h for hybridization. After two washes for 20 min each at 58°C, the sections were incubated with RNase buffer at room temperature for 5 min. Subsequently, the hybridized sections were treated with 10 μg/ml RNase A (Toyobo) in 10 mM Tris–HCl (pH 8.0), 0.5 M NaCl, and 1 mM EDTA for 30 min at 37°C. The sections were washed twice for 10 min each with 2 × SSC and 0.1% (w/v) NLS at 37°C and twice for 10 min each with 0.2 × SSC and 0.1% (w/v) NLS at 37°C. Sections were incubated with the blocking reagent at room temperature for 1 h. Then, the sections were incubated with a mixture of peroxidase-conjugated anti-digoxigenin antibodies (11207733910, 1:2000, Roche, Switzerland) and rabbit anti-RFP antibodies (600-401-379, 1:1000, Rockland, USA) at room temperature overnight. After three washes, the sections were incubated with a mixture of Alexa Fluor 488-conjugated streptavidin to visualize the riboprobes (S32354, 1:500, Invitrogen, USA) and Alexa Fluor 594-conjugated anti-rabbit IgG to amplify the virus (A-11012, 1:500, Invitrogen, USA) at room temperature for 4 h. Finally, all sections were incubated with Hoechst (ab228551, 1:1000, Abcam, USA) for 10 min at room temperature. All sections were air-dried and cover-slipped with a fluorescent mounting medium.

### Quantification and statistical analysis

All statistical data were shown as the mean ± SEM and were performed in Prism v.8.0 (GraphPad Software, Inc.) and statistical product service solutions (SPSS) v.21.0. At beginning, Shapiro–Wilk test was used to examine the normality test, and Levene’s test was used to examine homogeneity of variance test. Datasets that satisfied both the normality test and the homogeneity of variance test were analyzed with a parametric test. Datasets that satisfied the normality test but not the homogeneity of variance test were analyzed with a separate variance estimation *t*-test. Other data used non-parametric test. Parametric test contained two-tailed paired *t*-test and One-factor ANOVA here. Non-parametric test contained Mann–Whitney *U* test, Friedman’s *M* test and Kruskal–Wallis *H* test here. All statistical analyses were listed in the figure legends and were presented in Supplementary Table 1. *p < 0.05, ^**^p < 0.01, ^***^p < 0.001 and ^****^p < 0.0001.

## Results

### MK-801 administration caused schizophrenia-like behaviors in mice

**FIGURE 1:**
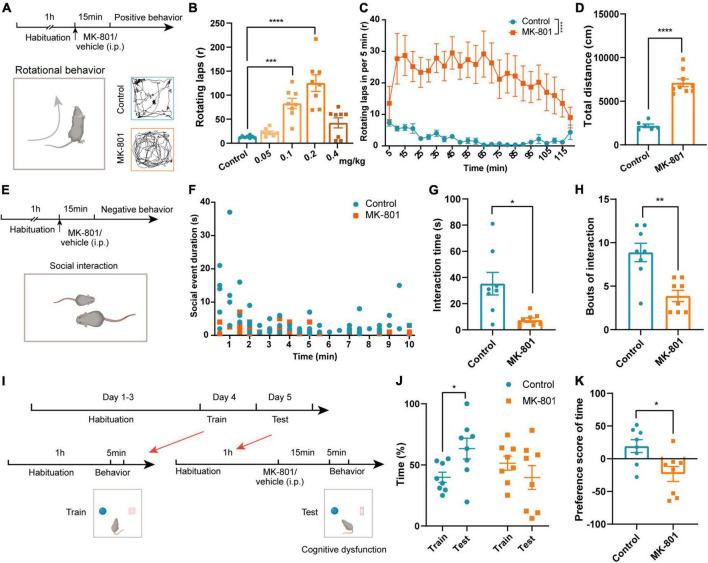
MK-801 caused schizophrenia-like behaviors in mice. **(A)** Timeline of the behavioral tests and diagram of the rotational behavior. **(B)** Laps of the rotational behavior produced by the injection of different concentrations of MK-801 (Kruskal–Wallis *H* test, Control *n* = 8 mice, MK-801 0.05 mg/kg *n* = 8 mice, MK-801 0.1 mg/kg *n* = 8 mice, MK-801 0.2 mg/kg *n* = 8 mice, MK-801 0.4 mg/kg *n* = 8 mice). **(C)** Rotational behavior was initiated 5 min after the injection and peaked at approximately 10–15 min, and the effects lasted approximately 2 h (Friedman’s *M* test, Control *n* = 6 mice, MK-801 *n* = 6 mice). **(D)** The total distance traveled by the MK-801-treated group and control group in the open field test (OFT) (unpaired *t*-test, Control *n* = 6 mice, MK-801 *n* = 9 mice). **(E–H)** Timeline of the behavioral tests and diagram of the social interaction test **(E)**. The social event duration in per minute **(F)**, interaction time [**(G)**, unpaired separate variance estimation *t*-test] and bouts of the interaction [**(H)**, Mann–Whitney *U* test] of MK-801-injected mice and control mice (Control *n* = 8 mice, MK-801 *n* = 8 mice). **(I–K)** Timeline of the behavioral tests and diagram of the novel object recognition (NOR) test **(I)**. The time spent with novel objects [**(J)**, paired *t*-test/Wilcoxon signed-rank test] and the preference score [**(K)**, unpaired *t*-test] of MK-801-injected mice and control mice (Control *n* = 8 mice, MK-801 *n* = 8 mice). Data are shown as the means ± SEM. *Indicates a significant difference between groups. *p < 0.05, **p < 0.01, ***p < 0.001, and ****p < 0.0001.

Patients with schizophrenia exhibit positive symptoms, negative symptoms, and cognitive deficits (Winship et al., 2019). We thus examined whether MK-801 administration could mimic these core symptoms in our animals. Previous study has showed that MK-801 injection caused stereotypic rotational behavior in C57BL/6 mice (Qi et al., 2008). Therefore, we firstly examined the concentration dependence of MK-801 inducing schizophrenia-like behaviors in mice according to rotational behavior (Figure 1A). Stereotypic rotational behavior was observed in mice after an intraperitoneal injection of different concentrations of MK-801 (0.05 mg/kg, 0.1 mg/kg, 0.2 mg/kg, or 0.4 mg/kg). And 0.2 mg/kg MK-801 injection induced a significant increase in the number of rotating laps and total distances that mice traveled (Figure 1B and Supplementary Figure 1, Control vs. MK-801 0.05 mg/kg: *p* = 0.207; Control vs. MK-801 0.1 mg/kg: *p* < 0.001; Control vs. MK-801 0.2 mg/kg: *p* < 0.0001; Control vs. MK-801 0.4 mg/kg: *p* = 0.143). Rotational behavior was initiated 5 min after the MK-801 injection and peaked at approximately 10–15 min, and the effects lasted approximately 2 h (Figure 1C, *p* < 0.0001). Therefore, we chose a concentration of 0.2 mg/kg MK-801 and 15 min injection interval for testing schizophrenia-like behaviors. In the open field test (OFT), the total distance traveled by the MK-801 group was increased significantly compared with that of the control group (Figure 1D, *t* = −8.601, *p* < 0.0001), which further indicated that MK-801 caused hyperactivity resembling the positive behavior of schizophrenia in mice. Patients with schizophrenia also show obvious apathy and social withdrawal (Dziwota et al., 2018). Indeed, the interaction time (*t* = 3.153, *p* = 0.015) and bouts of the interaction (*Z* = −2.907, *p* = 0.004) of MK-801-injected mice with the same homogeneous male mice were significantly reduced in the social interaction test (Figures 1E–H). Next, we examined the effects of MK-801 on learning and memory in mice (Figure 1I) using a novel object recognition (NOR) test. The results showed that control mice spent more time exploring new objects in the test sessions, while MK-801-injected mice were unable to distinguish the novel objects and familiar objects (Figure 1J, Control: *t* = 2.394, *p* = 0.048, paired *t*-test; MK-801: *Z* = −1.540, *p* = 0.123, Wilcoxon signed-rank test). The preference score of time in the MK-801 group was significantly decreased compared with control mice (Figure 1K, *t* = 2.837, *p* = 0.013), suggesting that their cognitive function was impaired. In addition, we also performed gait test to explore whether MK-801 caused other concomitant symptoms (such as dysfunction in locomotor coordination) in mice (Supplementary Figure 2A). The stand time, swing time, step cycle, and print width of MK-801-injected mice were decreased compared with those of control mice, while the swing speed, body speed, and print length were increased. The stride length showed no obvious change (Supplementary Figures 2B–I). Taken together, these results indicated that MK-801 produced several typical schizophrenia-like behaviors in mice.

### The ACC and BLA were involved in schizophrenia-like behaviors induced by MK-801

**FIGURE 2:**
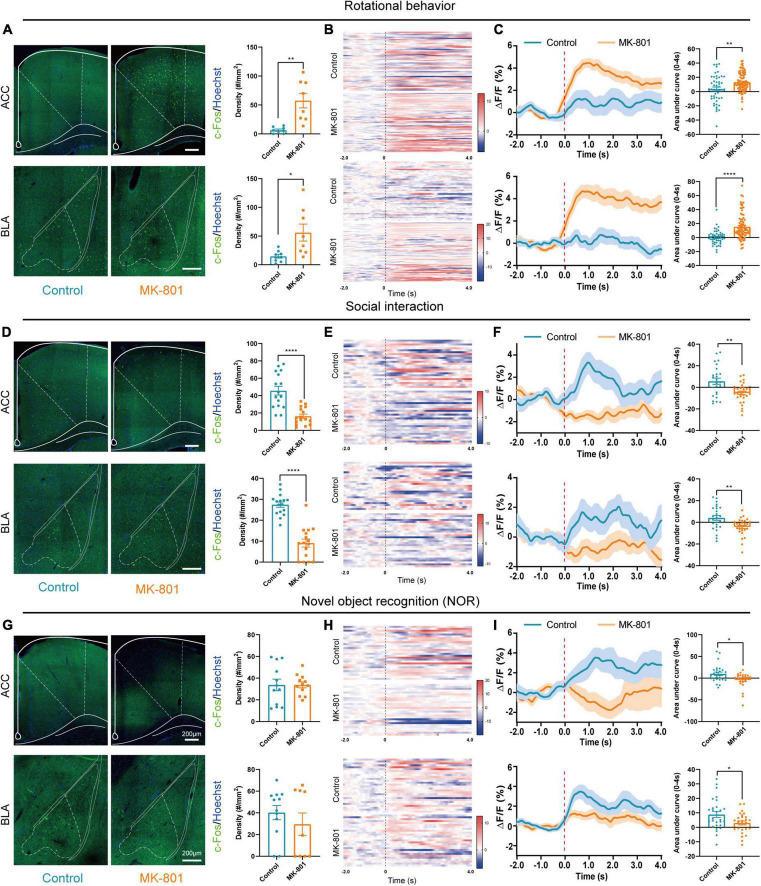
The anterior cingulate cortex (ACC) and basolateral amygdala (BLA) regions were affected by the MK-801 injection. **(A)** The c-Fos-positive cells in the ACC (unpaired separate variance estimation *t*-test, Control *n* = 8 slices/4 mice, MK-801 *n* = 8 slices/4 mice) and BLA (unpaired separate variance estimation *t*-test, Control *n* = 9 slices/4 mice, MK-801 *n* = 8 slices/4 mice) of the control and MK-801-treated mice after rotational behavior test. Scale bar, 200 μm. **(B,C)** Heatmaps showing Ca^2+^ signals aligned to the onset of rotational behavior. Each row represents a trial **(B)**. Representative traces of the averaged Ca^2+^ signals and the area under the curve of the Ca^2+^ signal in ACC (unpaired separate variance estimation *t*-test, Control *n* = 48 trails/4 mice, MK-801 *n* = 82 trails/4 mice) and BLA (Mann–Whitney *U* test, Control *n* = 48 trails/4 mice, MK-801 *n* = 82 trails/4 mice) **(C)**. **(D)** The c-Fos-positive cells in the ACC (unpaired separate variance estimation *t*-test, Control *n* = 18 slices/4 mice, MK-801 *n* = 16 slices/4 mice) and BLA (unpaired *t*-test, Control *n* = 16 slices/4 mice, MK-801 *n* = 16 slices/4 mice) of the control and MK-801-treated mice after social interaction test. Scale bar, 200 μm. **(E,F)** Heatmaps showing Ca^2+^ signals aligned to the onset of social interaction. Each row represents a trial **(E)**. Representative traces of the averaged Ca^2+^ signals and the area under the curve of the Ca^2+^ signal in ACC (unpaired separate variance estimation *t*-test, Control *n* = 25 trails/5 mice, MK-801 *n* = 28 trails/5 mice) and BLA (unpaired separate variance estimation *t*-test, Control *n* = 25 trails/5 mice, MK-801 *n* = 28 trails/5 mice) **(F)**. **(G)** The c-Fos-positive cells in the ACC (unpaired separate variance estimation *t*-test, Control *n* = 12 slices/4 mice, MK-801 *n* = 12 slices/4 mice) and BLA (Mann–Whitney *U* test, Control *n* = 12 slices/4 mice, MK-801 *n* = 9 slices/4 mice) of the control and MK-801-treated mice after novel object recognition (NOR) test. Scale bar, 200 μm. **(H,I)** Heatmaps showing Ca^2+^ signals aligned to the onset of novel object recognition. Each row represents a trial **(H)**. Representative traces of the averaged Ca^2+^ signals and the area under the curve of the Ca^2+^ signal in ACC (Mann–Whitney *U* test, Control *n* = 28 trails/4 mice, MK-801 *n* = 26 trails/4 mice) and BLA (unpaired separate variance estimation *t*-test, Control *n* = 28 trails/4 mice, MK-801 *n* = 26 trails/4 mice) **(I)**. Data are shown as the means ± SEM. *Indicates a significant difference between groups. *p < 0.05, **p < 0.01, and ****p < 0.0001.

According to previous studies, several structures including limbic, frontal, temporal, and subcortical areas were involved in pathogenesis of schizophrenia, and ACC and BLA were commonly reported to show abnormal structure and function in patients with schizophrenia (Pantelis et al., 2005; Fornito et al., 2009; Zeeb and Winstanley, 2011). To clarify the roles of ACC and BLA in MK-801 induced schizophrenia mouse model, we then evaluated the activation of the ACC and BLA by c-Fos staining and *in vivo* fiber photometry recording. The results showed that the density of c-Fos-positive cells was increased both in the ACC and BLA regions in the MK-801 group after rotational behaviors (Figure 2A, ACC: *t* = −4.034, *p* = 0.005; BLA: *t* = −2.732, *p* = 0.027). Similar to the results of c-Fos staining, the dynamic calcium signals of ACC and BLA neurons were significantly increased within seconds of rotational behavior onset in the MK-801-injected mice, and few differences were observed in the control mice (Figure 2B). The area under the curve was also significantly increased in MK-801-injected mice compared with control mice (Figure 2C, ACC: *t* = −3.076, *p* = 0.003; BLA: *Z* = −5.519, *p* < 0.0001). In contrast, the density of c-Fos-positive cells in the ACC and BLA of the MK-801-injected mice was significantly decreased compared with that in the control mice in the social interaction test (Figure 2D, ACC: *t* = 5.861, *p* < 0.0001; BLA: *t* = 8.103, *p* < 0.0001). The neuronal activities of the ACC and BLA in the MK-801 group also displayed an obvious decrease compared with the control group based on *in vivo* fiber photometry recording of the GCaMP6 signal (Figure 2E). Similarly, the area under the curve for the Ca^2+^ signal was decreased in MK-801-treated mice (Figure 2F, ACC: *t* = 3.248, *p* = 0.002; BLA: *t* = 3.088, *p* = 0.004). Finally, we examined c-Fos expression in these two brain regions after the NOR test, and the results showed no significant difference between the control mice and MK-801-injected mice (Figure 2G, ACC: *t* = 0.001, *p* = 0.999; BLA: *Z* = −0.288, *p* = 0.774). Changes in the calcium signals in the ACC and BLA of the MK-801 group were lower than those in the control group (Figure 2H), and the area under the curve for the Ca^2+^ signal was significantly decreased in the MK-801-injected mice (Figure 2I, ACC: *Z* = −2.441, *p* = 0.015; BLA: *t* = 2.411, *p* = 0.020). For all mice in the c-Fos staining (Supplementary Figures 3A–F) and in the fiber photometry recording tests (Supplementary Figures 3G–L), behavior test was examined simultaneously and the results showed similar abnormalities (mentioned in Figure 1) in MK-801-treated group. The sites of fiber photometry recording were also examined to be correct (Supplementary Figure 3M). In summary, we speculated that the abnormal neuronal dynamics in the ACC and BLA may be involved in the social withdrawal, stereotypic activity, and cognitive impairment induced by MK-801 administration.

### Chemogenetic regulation of BLA-ACC projecting neurons affected social and cognitive deficits but not stereotypic rotational behavior in MK-801-treated mice

**FIGURE 3:**
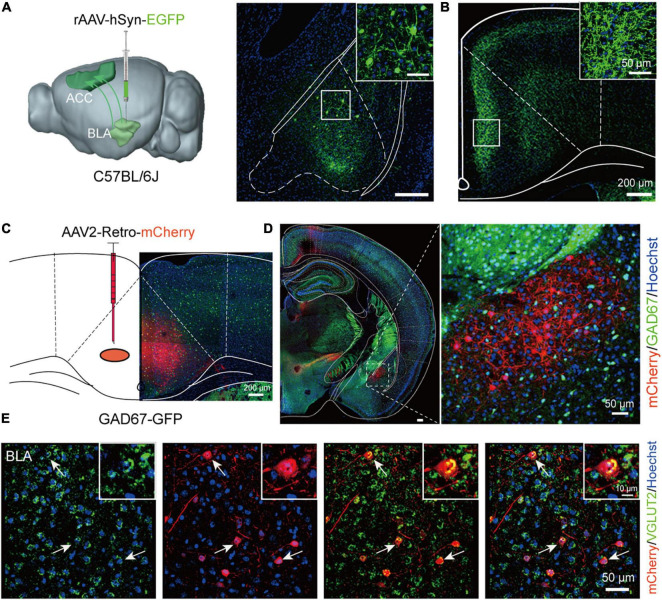
Basolateral amygdala (BLA) neurons send glutamatergic projections to the anterior cingulate cortex (ACC). **(A)** Schematic of a sagittal section of the mouse brain showing the injection of the rAAV-hSyn-EGFP virus into the BLA of C57BL/6J mice. **(B)** The ACC region received a large number of projection fiber terminals from the BLA. Scale bar, 200 μm. **(C)** Schematic of a coronal section of the mouse brain showing the injection of the AAV2-Retro-mCherry virus into the ACC of GAD67-GFP mice. Scale bar, 200 μm. **(D)** The cell bodies of BLA neurons showed red fluorescence after retrograde tracing from the ACC. Scale bar, 50 μm. **(E)** The *in situ* hybridization results showed that the *Vglut2* probe was colabeled with mCherry-positive neurons in the BLA (The white arrow showed the typically colabeled mCherry positive neurons with *Vglut2*). Scale bar, 50 μm.

Since both the BLA and ACC were involved in the schizophrenia-like behaviors induced by MK-801, we then wondered whether the BLA-ACC circuit modulated these behaviors. Before modulating this circuit, we first explored the properties of BLA-ACC projections. We injected rAAV-hSyn-EGFP-WPRE-pA into the BLA of C57BL/6J mice (Figure 3A) and identified a large number of projection fiber terminals in the ACC region (Figure 3B), which confirmed the projections from the BLA to the ACC. Then, we injected rAAV2/retro-hSyn-Cre-mCherry-WPRE-hGH pA into the ACC of GAD67-GFP mice to further resolve the connection between the BLA and ACC (Figure 3C). After 21 days of virus expression, we found that the cell bodies of BLA neurons exhibited red fluorescence, which verified that the ACC received projections from the BLA. We also found that retrolabeled neurons of the BLA were not colabeled with GAD-positive neurons (Figure 3D). Further *in situ* hybridization of the *Vglut2* probe showed that mCherry-positive neurons were colabeled with *Vglut2* (Figure 3E), suggesting that neurons of the BLA sending projections to the ACC were glutamatergic neurons.

**FIGURE 4:**
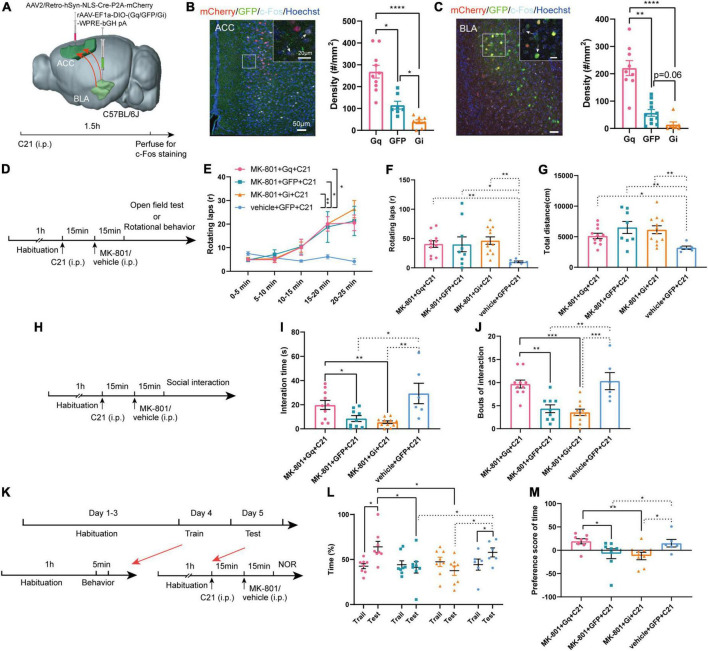
Chemogenetic regulation of basolateral amygdala-anterior cingulate cortex (BLA-ACC) projecting neurons affected social and cognitive deficits but not stereotypic rotational behavior in MK-801-treated mice. **(A)** Schematic of the mouse brain showing that the AAV2/Retro-hSyn-NLS-Cre-P2A-mCherry virus was bilaterally injected into the ACC and the rAAV-EF1a-DIO-hM3Dq (Gq)/GFP/hM4Di (Gi)-WPRE-bGH pA viruses were bilaterally delivered into the BLA of C57BL/6J mice. **(B,C)** The c-Fos-positive cells in the ACC [**(C)**, Kruskal–Wallis *H* test, Gq *n* = 10 slices/6 mice, GFP *n* = 7 slices/6 mice, Gi *n* = 8 slices/6 mice] and BLA [**(C)**, Kruskal–Wallis *H* test, Gq *n* = 9 slices/6 mice, GFP *n* = 11 slices/6 mice, Gi *n* = 7 slices/6 mice] after C21 injection. **(D)** Timeline of chemogenetic regulation of rotational behavior and OFT. **(E–G)** The results of the rotational behavior test [**(E)**, Friedman’s *M* test; **(F)**, Kruskal–Wallis *H* test, MK-801 + Gq + C21 *n* = 11 mice, MK-801 + GFP + C21 *n* = 9 mice, MK-801 + Gi + C21 *n* = 12 mice, vehicle + GFP + C21 *n* = 6 mice] and OFT [**(G)**, Kruskal–Wallis *H* test, MK-801 + Gq + C21 *n* = 11 mice, MK-801 + GFP + C21 *n* = 8 mice, MK-801 + Gi + C21 *n* = 12 mice, vehicle + GFP + C21 *n* = 6 mice] after chemogenetic regulation of BLA-ACC circuit. **(H)** Timeline of chemogenetic regulation of social interaction behavior. **(I,J)** The results of the social interaction test (Kruskal–Wallis *H* test/One-factor ANOVA, MK-801 + Gq + C21 *n* = 10 mice, MK-801 + GFP + C21 *n* = 9 mice, MK-801 + Gi + C21 *n* = 11 mice, vehicle + GFP + C21 *n* = 6 mice) after chemogenetic regulation of BLA-ACC circuit. **(K)** Timeline of chemogenetic regulation of NOR test. **(L,M)** The results of the percentage of time that mice spent in exploring the novel object [**(L)**, One-factor ANOVA] and the preference scores of NOR test after chemogenetic regulation of BLA-ACC circuit [**(M)**, Kruskal–Wallis *H* test, MK-801 + Gq + C21 *n* = 8 mice, MK-801 + GFP + C21 *n* = 8 mice, MK-801 + Gi + C21 *n* = 8 mice, vehicle + GFP + C21 *n* = 6 mice]. Data are shown as the means ± SEM. *Indicates a significant difference between groups. *p < 0.05, **p < 0.01, ***p < 0.001, and ****p < 0.0001.

Next, we examined whether specifically regulated BLA-ACC projection neurons modulated MK-801-induced behavioral dysfunction. A series of rAAV-EF1a-DIO-hM3Dq (Gq)/GFP/hM4Di (Gi)-WPRE-bGH pA viruses were bilaterally delivered into the BLA, and an AAV2/Retro-hSyn-NLS-Cre-P2A-mCherry virus was bilaterally injected into the ACC by stereotactic injection (Figure 4A). This approach allowed us to specifically express DREADD proteins on a group of BLA projection neurons that send fibers to innervate the ACC. Three weeks after the virus was injected, we injected the C21 intraperitoneally 30 min before the behavior tests to selectively activate or inhibit the BLA-ACC projection neurons. Meanwhile, MK-801 was intraperitoneally injected 15 min before the test (Figures 4D, H, K). The c-Fos^+^ cell density was increased in the Gq group but decreased in the Gi group compared with GFP control mice after the mice were treated with C21 (Figures 4B, C, c-Fos of ACC: Gq vs. GFP, *p* = 0.029, Gq vs. Gi, *p* < 0.0001, GFP vs. Gi, *p* = 0.044, Kruskal–Wallis *H* test; c-Fos of BLA: Gq vs. GFP, *p* = 0.005, Gq vs. Gi, *p* < 0.0001, GFP vs. Gi, *p* = 0.058, Kruskal–Wallis *H* test), indicating that the viral strategy was effective. However, in the positive behavior tests, neither the stereotypic rotational behavior nor the hyperactivity of the mice was altered by chemogenetic regulation (Figures 4D–G). In social interaction tests (Figure 4H), the mice in the Gq-MK-801 group displayed an increased interaction time (Figure 4I, MK-801 + Gq + C21 vs. MK-801 + GFP + C21, *p* = 0.029, MK-801 + Gq + C21 vs. MK-801 + Gi + C21, *p* = 0.003, Kruskal–Wallis *H* test) and bouts compared with the stranger mouse (Figure 4J, MK-801 + Gq + C21 vs. MK-801 + GFP + C21, *p* = 0.002, MK-801 + Gq + C21 vs. MK-801 + Gi + C21, *p* < 0.001, One-factor ANOVA). Although a significant difference in interaction time and bouts were not observed between the Gi-MK-801 and GFP-MK-801 mice, the Gi-MK-801 group also showed a significant reduction compared with the Gq-MK-801 group, which may be related to the “floor effect.” Last, we found that the Gq-MK-801 group spent more time exploring the novel object after activating the BLA-ACC circuit by C21 injection in the NOR test (Figures 4K, L, MK-801 + Gq + C21: Train vs. Test, *p* = 0.014, paired *t*-test; Test: MK-801 + Gq + C21 vs. MK-801 + GFP + C21, *p* = 0.039, One-factor ANOVA). However, no significant difference was observed in the Gi-MK-801 mice (Figure 4L, Gi-MK-801: Train vs. Test, *p* = 0.194, paired *t*-test; Test: MK-801 + Gi + C21 vs. MK-801 + GFP + C21, *p* = 0.964, One-factor ANOVA) and GFP-MK-801 mice (Figure 4L, MK-801 + GFP + C21: Train vs. Test, *p* = 0.999, Wilcoxon signed-rank test) after C21 was injected. The preference score of Gq-MK-801 mice was also significantly higher than that of Gi-MK-801 and GFP-MK-801 mice (Figure 4M, MK-801 + Gq + C21 vs. MK-801 + GFP + C21, *p* = 0.027, MK-801 + Gq + C21 vs. MK-801 + Gi + C21, *p* = 0.003, MK-801 + GFP + C21 vs. MK-801 + Gi + C21, *p* = 0.461, Kruskal–Wallis *H* test). Interestingly, we also found that inhibition of BLA-ACC pathway could reduce the social interaction time and time spent in novel objects but not rotational behavior in vehicle injected-mice (Supplementary Figures 4A–G). Gait abnormalities were not regulated by this circuit (Supplementary Figures 5A–H). Based on the final site confirmation, we only included the behavioral data from mice in which the virus was injected in the correct site (Supplementary Figure 6). Taken together, the social disorder and cognitive dysfunction of mice caused by MK-801 were alleviated by specifically regulating the BLA-ACC neural circuit, but the regulation of this circuit had no effect on schizophrenia-like positive behaviors and gait dysfunction.

### Optogenetic modulation of BLA-ACC projecting neurons exerted a similar effect to chemogenetic regulation

**FIGURE 5:**
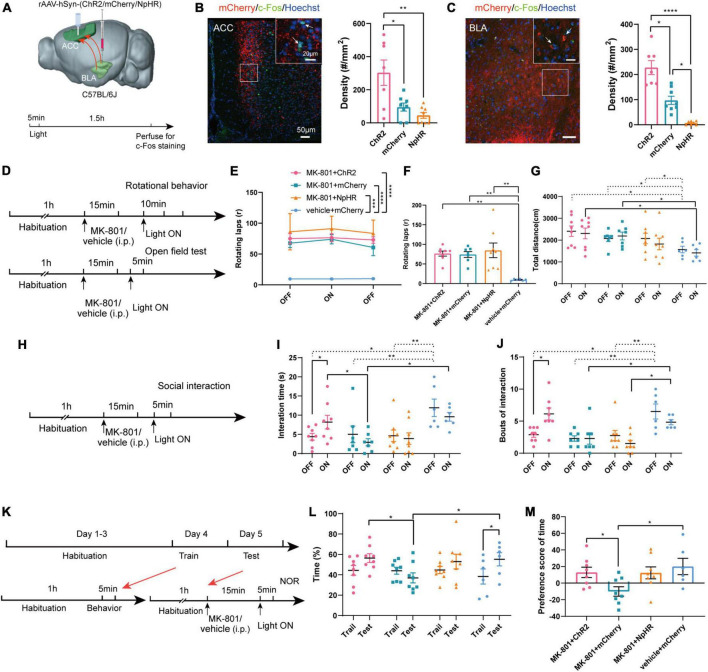
Optogenetic modulation of basolateral amygdala-anterior cingulate cortex (BLA-ACC) projecting neurons affected social and cognitive deficits but not stereotypic rotational behavior in MK-801-treated mice. **(A)** Schematic of the mouse brain showing the bilateral injection of the rAAV2-hSyn-(ChR2/mCherry/NpHR)-WPRE-pA virus into the BLA and optical fibers implanted into the ACC of C57BL/6J mice. **(B,C)** The c-Fos-positive cells in the ACC [**(B)**, Kruskal–Wallis *H* test, ChR2 *n* = 7 slices/4 mice, mCherry *n* = 8 slices/4 mice, NpHR *n* = 8 slices/4 mice] and BLA [**(C)**, Kruskal–Wallis *H* test, ChR2 *n* = 7 slices/4 mice, mCherry *n* = 8 slices/4 mice, NpHR *n* = 7 slices/4 mice] after laser light on. **(D)** Timeline of optogenetic regulation of rotational behavior and OFT. **(E–G)** The results of the rotational behavior test [**(E)**, Friedman’s *M* test; **(F)**, Kruskal–Wallis *H* test, MK-801 + ChR2 *n* = 8 mice, MK-801 + mCherry *n* = 7 mice, MK-801 + NpHR *n* = 8 mice, vehicle + mCherry *n* = 6 mice] and OFT [**(G)**, One-factor ANOVA, MK-801 + ChR2 *n* = 8 mice, MK-801 + mCherry *n* = 7 mice, MK-801 + NpHR *n* = 8 mice, vehicle + mCherry *n* = 6 mice] after optogenetic regulation of BLA-ACC circuit. **(H)** Timeline of optogenetic regulation of social interaction behavior. **(I,J)** The results of the social interaction test (Kruskal–Wallis *H* test/One-factor ANOVA, MK-801 + ChR2 *n* = 8 mice, MK-801 + mCherry *n* = 7 mice, MK-801 + NpHR *n* = 8 mice, vehicle + mCherry *n* = 6 mice) after optogenetic regulation of BLA-ACC circuit. **(K)** Timeline of optogenetic regulation of NOR test. **(L,M)** The results of the percentage of time that mice spent in exploring the novel object [**(L)**, One-factor ANOVA] and the preference scores of NOR test after optogenetic regulation of BLA-ACC circuit [**(M)**, One-factor ANOVA, MK-801 + ChR2 *n* = 8 mice, MK-801 + mCherry *n* = 8 mice, MK-801 + NpHR *n* = 8 mice, vehicle + mCherry *n* = 6 mice]. Data are shown as the means ± SEM. *indicates a significant difference between groups. *p < 0.05, **p < 0.01, ***p < 0.001, and ****p < 0.0001.

Compared with chemogenetics, optogenetics has higher temporal resolution. We next used optogenetic methods to further verify the effects of the BLA-ACC neural circuit on mouse behavior induced by MK-801. To specifically regulate the BLA-ACC neural circuit, we injected ChR2/mCherry/NpHR into the BLA and implanted fiber-optic probes bilaterally in the ACC. Optical stimulation with blue light (473 nm, 5 ms, 20 Hz, 10 mW/mm^2^) and optical inhibition with yellow light (594 nm, constant, 10 mW/mm^2^) were applied to activate or inhibit the BLA-ACC projection neurons during the behavioral test (Figure 5A). The application of 20 Hz blue light induced much higher c-Fos expression in both the ACC and BLA of the ChR2 group than in the mCherry group. Yellow light produced an almost opposite effect on both ACC and BLA in the NpHR group (Figures 5B, C, c-Fos of ACC: ChR2 vs. mCherry, *p* = 0.047, ChR2 vs. NpHR, *p* = 0.005, mCherry vs. NpHR, *p* = 0.236, Kruskal–Wallis *H* test; c-Fos of BLA: ChR2 vs. mCherry, *p* = 0.046, ChR2 vs. NpHR, *p* < 0.0001, mCherry vs. NpHR, *p* = 0.019, Kruskal–Wallis *H* test). In the positive behavior tests, neither the stereotypic rotational behavior nor the hyperactivity of the mice was regulated by blue or yellow light stimulation (Figures 5D–G). Regarding to other schizophrenia-like behaviors, the interaction time (Figures 5H, I, MK-801 + ChR2: OFF vs. ON, *p* = 0.043, paired *t*-test) and bouts of interaction (Figure 5J, MK-801 + ChR2: OFF vs. ON, *p* = 0.013, paired *t*-test) were significantly increased when the light was on in the ChR2-MK-801 group compared with the light off phase. The social interaction time (Figure 5H, ON: MK-801 + ChR2 vs. MK-801 + mCherry, *p* = 0.047, One-factor ANOVA) and bouts (Figure 5I, ON: MK-801 + ChR2 vs. MK-801 + mCherry, *p* = 0.007, One-factor ANOVA) of ChR2 mice were also increased compared with those in the mCherry-MK-801 group during the light-on period. The yellow light stimulation did not change the interaction time (Figure 5I, MK-801 + NpHR: OFF vs. ON, *p* = 0.527, paired *t*-test; ON: MK-801 + NpHR vs. MK-801 + mCherry, *p* = 0.960, One-factor ANOVA) and bouts of interaction (Figure 5I, MK-801 + NpHR: OFF vs. ON, *p* = 0.074, Wilcoxon signed-rank test; ON: MK-801 + NpHR vs. MK-801 + mCherry, *p* = 0.880, One-factor ANOVA) in the NpHR-MK-801 mice, which may be related to the “floor effect.” The results of the NOR test were similar to that in the chemogenetic regulation, the activation of BLA-ACC projection neurons with blue light increased the time spent exploring the novel object (Figures 5K, L, ON: MK-801 + ChR2 vs. MK-801 + mCherry, *p* = 0.042, One-factor ANOVA) and the preference score (Figure 5M, ON: MK-801 + ChR2 vs. MK-801 + mCherry, *p* = 0.018, One-factor ANOVA) compared with the mCherry-injected mice. Although there was no statistical difference in the time spent exploring the novel object after blue light activation this pathway, there was an obvious increase trend in the MK-801 + ChR2 group (Figure 5L, MK-801 + ChR2: Train vs. Test, *p* = 0.080, paired *t*-test). The finding that the time mice spent exploring novel objects after yellow light-mediated inhibition of the circuit was not altered compared with before light exposure and even showed an increasing trend is confusing. But in vehicle injected mice, we found that inhibition of BLA-ACC pathway could reduce the time spent exploring novel objects of mice, and we also observed that activation of BLA-ACC pathway could increase the social interaction time and time spent in novel objects but not rotational behavior (Supplementary Figures 4H–N). Based on the final site confirmation, we only included the behavioral data from mice with the correct virus injection and optical implantation sites (Supplementary Figure 7). Based on these results, the social dysfunction of mice caused by MK-801 was alleviated by specifically regulating the BLA-ACC neural circuit, and the cognitive dysfunction was partially affected by the BLA-ACC circuit, but no effect on schizophrenia-like positive behaviors was observed.

## Discussion

In the present study, we focused on the roles of neural circuitry in schizophrenia-like behaviors. We used the non-competitive NMDA receptor antagonist MK-801 to induce schizophrenia-like behaviors such as stereotypic rotational behavior and hyperlocomotion, social interaction defects and cognitive dysfunction in the NOR test. c-Fos staining and fiber photometry indicated the involvement of ACC and BLA brain regions in these schizophrenia-like behaviors. Furthermore, the activation of the BLA-ACC neural circuit with both chemogenetic and optogenetic methods alleviated social and cognitive deficits but not stereotypic rotational behavior in the mouse model of MK-801-induced schizophrenia. The present findings not only highlight the potential roles of the BLA-ACC circuit in improving cognitive and social impairments but also might provide important insights into the treatment of schizophrenia in the future.

Schizophrenia is still one of the most severe and lethal mental disorders, imposing substantial human suffering and societal expenditure. Therefore, although animal models face the main difficulty of replicating symptoms, they are crucial tools to study pathological changes associated with schizophrenia (Adell et al., 2012). In recent years, more than twenty different animal models of schizophrenia have been developed (Carpenter and Koenig, 2008), and all these available models fit into four categories: developmental, genetic manipulation, drug-induced, and lesion. Based on these animal models, the dopamine dysfunction theory became one of the major hypotheses for the pathophysiology of schizophrenia. First-generation and second-generation antipsychotics were developed according to this theory. However, most of these drugs were only effective at treating the positive symptoms, while negative symptoms and cognitive impairments were not sufficiently improved (van Os and Kapur, 2009). The hypothesis of NMDA receptor hypofunction emerged as a new important hypothesis since a single subanesthetic dose of non-competitive NMDA antagonists leads to schizophrenia-like symptoms in healthy individuals in the clinic (Javitt and Zukin, 1991). Acute administration of MK-801 became a desired model of schizophrenia, especially for the study of negative and cognitive impairments. Here, we confirmed that the administration of MK-801 to mice induced positive behaviors (stereotypic rotating behavior and increased distance traveled in the OFT), negative behaviors (social interaction deficits), and cognitive deficits (decreased preference score in the NOR test). These findings agree with existing studies in the literature (Wu et al., 2005; Moy et al., 2013; Cieslik et al., 2019; Murueta-Goyena et al., 2019). Interestingly, we discovered that mice treated with MK-801 exhibited gait dysfunction, consistent with a previous study showing that NMDA receptor activation might be important for locomotor coordination (McEwen et al., 1999). Moreover, although the acute MK-801 model may not be suitable for exploring the whole pathophysiological progress of schizophrenia, considering its high face validity, the acute MK-801 model may be a powerful tool to understand the neural circuit mechanism underlying schizophrenia-like symptoms.

Currently, progress has been achieved in understanding which brain regions are critical for the effect of NMDA antagonists. Several different regions, including the dorsolateral prefrontal cortex, the rostral ACC, the hippocampal formation, and the BLA, are involved in disturbances in individuals with schizophrenia. Postmortem studies have revealed dysfunction of the gamma-aminobutyric acid (GABA) and glutamate systems in these regions (Benes, 2010). An excess amount of incoming excitatory activity from other cortical or subcortical regions that project to superficial lamina may also play a role in the generation of subtle structural and/or functional anomalies (Benes, 1999). In the present study, acute administration of MK-801 induced a higher density of c-Fos expression and increased neuronal Ca^2+^ activity in both the ACC and BLA when mice exhibited stereotypic rotating behaviors. Meanwhile, a low density of c-Fos expression and decreased neuronal Ca^2+^ activity were observed when MK-801-treated mice exhibited social dysfunctions. The results of c-Fos staining and fiber photometry recordings were different seemed strange in NOR test. As mice also exhibited rotational behavior in the NOR test, we further compared c-Fos expression in MK-801-injected mice in the rotational behavior test (Figure 2A) and in the NOR test (Figure 2G). Interestingly, we observed lower c-Fos expression in the NOR test than the rotational behavior test (Supplementary Figures 8A, B, c-Fos of ACC: *t* = 2.243, *p* = 0.038; c-Fos of BLA: *Z* = −1.452, *p* = 0.146). The results indicated that rotational behavior in the NOR test might influence the c-Fos staining. However, the results of fiber photometry in NOR test showed that the calcium signals in the ACC and BLA of the MK-801 group were lower than those in the control group, which indicated that rotational behavior in the NOR test did not influence the results of fiber photometry recording. To sum up, these results indicated that mice treated with MK-801 indeed showed lower neural activation in the ACC and BLA than the control group in the NOR test. These phenomena are easy to understand separately. As shown in our previous studies, the ACC and BLA are activated after stereotypic behaviors (Liu et al., 2021), and ACC defects are causally linked to social dysfunction (Guo et al., 2019). Other lesion studies also suggested that the ACC was most important region for processing of cognition (Rudebeck et al., 2006). The relationship of the BLA with social behavior and cognition has been reported frequently as well (Felix-Ortiz and Tye, 2014; Yang and Wang, 2017). However, the results may be difficult to understand when we combine these data—both ACC and BLA neurons display opposite changes in activity when the “mice with schizophrenia-like behaviors” exhibit different symptoms. We cannot yet fully explain these findings. However, based on the behaviors we observed, we speculated that the “schizophrenia mouse model” may respond differently when faced with different external information inputs. For example, stereotypic rotating behavior was observed only in a small box (20 cm × 20 cm). We thought a small environment may easily trigger the “schizophrenia-like behaviors,” generating rotating behavior when the mice explored this area. Indeed, we did not observe obvious rotation in the MK-801 group in the OFT chamber (50 cm × 50 cm). In the social interaction test, although the size of the home cage was not large, the major information the subject was presented was social cues, including the odor, vision, and tactile sense from strangers. This interesting question will be explored in the future.

Experimental evidence indicates that anatomical and functional connections between the ACC and BLA play an important role in long-term withdrawal memory or decision-making tasks of cognitive effort (Hosking et al., 2014; Shao et al., 2021), indicating that defects in this circuit might participate in cognitive dysfunction in individuals with schizophrenia. Indeed, in the present study, chemogenetic and optogenetic activation of the BLA-ACC neural circuit alleviated social and cognitive deficits in MK-801-treated mice, and we illustrated that this circuit is an excitatory projection as well. However, modulation of this neural circuit failed to affect stereotypic rotational behavior and gait abnormalities. Converging lines of evidence from animal studies strongly suggest that MK-801 affects cortical function by reducing the function of NMDA receptors on GABAergic interneurons (Thomases et al., 2013), thereby further affecting cognitive function. For example, alterations in the parvalbumin-containing GABAergic interneuron number and function in the prefrontal cortex are strongly associated with schizophrenia (Gonzalez-Burgos et al., 2015). Other studies using double *in situ* hybridization on postmortem tissue showed a reduction in the expression of the NR2A subunit on parvalbumin-positive interneurons (Woo et al., 2004). Generally, these alterations are presumed to result in reduced output of these neurons, thereby disturbing circuit function. Lisman et al. (2008) considered that NMDAR hypofunction would be falsely “interpreted” as inactivity of pyramidal cells. As part of its homeostatic function, interneurons would then attempt to compensate for the apparent inactivity by reducing inhibitory output through a decrease GAD67 levels and a subsequent decrease in GABA production (Lisman et al., 2008). We thus presumed that activation of the BLA-ACC circuit might restore and reduce this compensatory activity. In summary, the present study provided novel evidence for the roles of the ACC and BLA in schizophrenia and argued that modulation of the BLA-ACC circuit played a critical role in regulating cortical activities that are essential for social behavioral and cognitive processes.

Our work also has some limitations. First, seven different cognitive domains are specifically relevant for schizophrenia (Young et al., 2009), however, we only examined working memory defects (NOR test) here. Second, while these tests provide a relatively high-throughput evaluation of symptom progression or alleviation with treatment, their relationship to human symptoms can be questioned (Young et al., 2009). Third, since MK-801 injection caused basic locomotion, rotation, and motor function dysfunction, it might influence the results of social interaction test and NOR test to some extent. Despite these shortcomings, future studies investigating pathological changes in the BLA-ACC circuit to improve other behavioral and cognitive deficits in individuals with schizophrenia are essential to increase our understanding of the neurobiological basis of the disorder and for the development of novel drugs with improved therapeutic efficacy.

## Data availability statement

The original contributions presented in this study are included in this article/Supplementary material, further inquiries can be directed to the corresponding authors.

## Ethics statement

This animal study was reviewed and approved by Institutional Animal Care and Use Committee (IACUC-20200356) of the Fourth Military Medical University.

## Author contributions

XH, HL, WW, and SW: conceptualization. XH, YL, and HL: methodology. XH, YL, HL, JX, BT, ZT, and HD: investigation. XH and HL: writing—original draft. WW and SW: writing—review and editing, funding acquisition, resources, and supervision. All authors read and approved the final manuscript.

## References

[B1] Adell A. Jimenez-Sanchez L. Lopez-Gil X. Romon T. (2012). Is the acute NMDA receptor hypofunction a valid model of schizophrenia? *Schizophr. Bull.* 38 9–14. 10.1093/schbul/sbr133 21965469 PMC3245580

[B2] Benes F. M. (1998). Model generation and testing to probe neural circuitry in the cingulate cortex of postmortem schizophrenic brain. *Schizophr. Bull.* 24 219–230. 10.1093/oxfordjournals.schbul.a033322 9613622

[B3] Benes F. M. (1999). Alterations of neural circuitry within layer II of anterior cingulate cortex in schizophrenia. *J. Psychiatr. Res.* 33 511–512. 10.1016/s0022-3956(99)00035-7 10628527

[B4] Benes F. M. (2010). Amygdalocortical circuitry in schizophrenia: From circuits to molecules. *Neuropsychopharmacology* 35 239–257. 10.1038/npp.2009.116 19727065 PMC3055447

[B5] Bryll A. Krzysciak W. Karcz P. Smierciak N. Kozicz T. Skrzypek J. (2020). The relationship between the level of anterior cingulate cortex metabolites, brain-periphery redox imbalance, and the clinical state of patients with schizophrenia and personality disorders. *Biomolecules* 10:1272. 10.3390/biom10091272 32899276 PMC7565827

[B6] Buck S. A. Quincy Erickson-Oberg M. Logan R. W. Freyberg Z. (2022). Relevance of interactions between dopamine and glutamate neurotransmission in schizophrenia. *Mol. Psychiatry* 27 3583–3591. 10.1038/s41380-022-01649-w 35681081 PMC9712151

[B7] Carpenter W. T. Koenig J. I. (2008). The evolution of drug development in schizophrenia: Past issues and future opportunities. *Neuropsychopharmacology* 33 2061–2079. 10.1038/sj.npp.1301639 18046305 PMC2575138

[B8] Cieslik P. Radulska A. Pelikant-Malecka I. Ploska A. Kalinowski L. Wieronska J. M. (2019). Reversal of MK-801-induced disruptions in social interactions and working memory with simultaneous administration of LY487379 and VU152100 in Mice. *Int. J. Mol. Sci.* 20:2781. 10.3390/ijms20112781 31174329 PMC6600181

[B9] Das P. Kemp A. H. Flynn G. Harris A. W. Liddell B. J. Whitford T. J. (2007). Functional disconnections in the direct and indirect amygdala pathways for fear processing in schizophrenia. *Schizophr. Res.* 90 284–294. 10.1016/j.schres.2006.11.023 17222539

[B10] Dziwota E. Stepulak M. Z. Wloszczak-Szubzda A. Olajossy M. (2018). Social functioning and the quality of life of patients diagnosed with schizophrenia. *Ann. Agric. Environ. Med.* 25 50–55. 10.5604/12321966.1233566 29575877

[B11] Felix-Ortiz A. C. Tye K. M. (2014). Amygdala inputs to the ventral hippocampus bidirectionally modulate social behavior. *J. Neurosci.* 34 586–595. 10.1523/JNEUROSCI.4257-13.2014 24403157 PMC3870937

[B12] Fornito A. Yucel M. Dean B. Wood S. J. Pantelis C. (2009). Anatomical abnormalities of the anterior cingulate cortex in schizophrenia: Bridging the gap between neuroimaging and neuropathology. *Schizophr. Bull.* 35 973–993. 10.1093/schbul/sbn025 18436528 PMC2728810

[B13] Gallant S. Welch L. Martone P. Shalev U. (2017). Effects of chronic prenatal MK-801 treatment on object recognition, cognitive flexibility, and drug-induced locomotor activity in juvenile and adult rat offspring. *Behav. Brain Res.* 328 62–69. 10.1016/j.bbr.2017.04.004 28390877

[B14] Garrick J. M. Costa L. G. Cole T. B. Marsillach J. (2021). Evaluating gait and locomotion in rodents with the catwalk. *Curr. Protoc.* 1:e220. 10.1002/cpz1.220 34370398 PMC8363132

[B15] Gonzalez-Burgos G. Cho R. Y. Lewis D. A. (2015). Alterations in cortical network oscillations and parvalbumin neurons in schizophrenia. *Biol. Psychiatry* 77 1031–1040. 10.1016/j.biopsych.2015.03.010 25863358 PMC4444373

[B16] Goutaudier R. Coizet V. Carcenac C. Carnicella S. (2020). Compound 21, a two-edged sword with both DREADD-selective and off-target outcomes in rats. *PLoS One* 15:e0238156. 10.1371/journal.pone.0238156 32946510 PMC7500623

[B17] Grotta J. Clark W. Coull B. Pettigrew L. C. Mackay B. Goldstein L. B. (1995). Safety and tolerability of the glutamate antagonist CGS 19755 (Selfotel) in patients with acute ischemic stroke. Results of a phase IIa randomized trial. *Stroke* 26 602–605. 10.1161/01.str.26.4.602 7709405

[B18] Guo B. Chen J. Chen Q. Ren K. Feng D. Mao H. (2019). Anterior cingulate cortex dysfunction underlies social deficits in Shank3 mutant mice. *Nat. Neurosci.* 22 1223–1234. 10.1038/s41593-019-0445-9 31332372

[B19] Hosking J. G. Cocker P. J. Winstanley C. A. (2014). Dissociable contributions of anterior cingulate cortex and basolateral amygdala on a rodent cost/benefit decision-making task of cognitive effort. *Neuropsychopharmacology* 39 1558–1567. 10.1038/npp.2014.27 24496320 PMC4023153

[B20] Howes O. McCutcheon R. Stone J. (2015). Glutamate and dopamine in schizophrenia: An update for the 21st century. *J. Psychopharmacol.* 29 97–115. 10.1177/0269881114563634 25586400 PMC4902122

[B21] Janak P. H. Tye K. M. (2015). From circuits to behaviour in the amygdala. *Nature* 517 284–292. 10.1038/nature14188 25592533 PMC4565157

[B22] Javitt D. C. Zukin S. R. (1991). Recent advances in the phencyclidine model of schizophrenia. *Am. J. Psychiatry* 148 1301–1308. 10.1176/ajp.148.10.1301 1654746

[B23] Jendryka M. Palchaudhuri M. Ursu D. van der Veen B. Liss B. Katzel D. (2019). Pharmacokinetic and pharmacodynamic actions of clozapine-N-oxide, clozapine, and compound 21 in DREADD-based chemogenetics in mice. *Sci. Rep.* 9:4522. 10.1038/s41598-019-41088-2 30872749 PMC6418145

[B24] Jones C. A. Watson D. J. Fone K. C. (2011). Animal models of schizophrenia. *Br. J. Pharmacol.* 164 1162–1194. 10.1111/j.1476-5381.2011.01386.x 21449915 PMC3229756

[B25] Lauriello J. (2020). Prevalence and impact of relapse in patients with schizophrenia. *J. Clin. Psychiatry* 81:MS19053BR1C. 10.4088/JCP.MS19053BR1C 32237296

[B26] Lisman J. E. Coyle J. T. Green R. W. Javitt D. C. Benes F. M. Heckers S. (2008). Circuit-based framework for understanding neurotransmitter and risk gene interactions in schizophrenia. *Trends Neurosci.* 31 234–242. 10.1016/j.tins.2008.02.005 18395805 PMC2680493

[B27] Liu H. Huang X. Li Y. Xi K. Han Y. Mao H. (2022). TNF signaling pathway-mediated microglial activation in the PFC underlies acute paradoxical sleep deprivation-induced anxiety-like behaviors in mice. *Brain Behav. Immun.* 100 254–266. 10.1016/j.bbi.2021.12.006 34915154

[B28] Liu H. Huang X. Xu J. Mao H. Li Y. Ren K. (2021). Dissection of the relationship between anxiety and stereotyped self-grooming using the Shank3B mutant autistic model, acute stress model and chronic pain model. *Neurobiol. Stress* 15:100417. 10.1016/j.ynstr.2021.100417 34815987 PMC8591549

[B29] McEwen M. L. Van Hartesveldt C. Stehouwer D. J. (1999). The NMDA antagonist, MK-801, alters L-DOPA-induced air-stepping in neonatal rats. *Brain Res. Dev. Brain Res.* 115 33–40. 10.1016/s0165-3806(99)00051-6 10366700

[B30] Moy S. S. Nonneman R. J. Shafer G. O. Nikolova V. D. Riddick N. V. Agster K. L. (2013). Disruption of social approach by MK-801, amphetamine, and fluoxetine in adolescent C57BL/6J mice. *Neurotoxicol. Teratol.* 36 36–46. 10.1016/j.ntt.2012.07.007 22898204 PMC3509253

[B31] Murueta-Goyena A. Morera-Herreras T. Miguelez C. Gutierrez-Ceballos A. Ugedo L. Lafuente J. V. (2019). Effects of adult enriched environment on cognition, hippocampal-prefrontal plasticity and NMDAR subunit expression in MK-801-induced schizophrenia model. *Eur. Neuropsychopharmacol.* 29 590–600. 10.1016/j.euroneuro.2019.03.009 30926324

[B32] Pantelis C. Yucel M. Wood S. J. Velakoulis D. Sun D. Berger G. (2005). Structural brain imaging evidence for multiple pathological processes at different stages of brain development in schizophrenia. *Schizophr. Bull.* 31 672–696. 10.1093/schbul/sbi034 16020551

[B33] Prestia A. Cavedo E. Boccardi M. Muscio C. Adorni A. Geroldi C. (2015). Hippocampal and amygdalar local structural differences in elderly patients with schizophrenia. *Am. J. Geriatr. Psychiatry* 23 47–58. 10.1016/j.jagp.2014.01.006 24534522 PMC4382088

[B34] Qi C. Zou H. Zhang R. Zhao G. Jin M. Yu L. (2008). Age-related differential sensitivity to MK-801-induced locomotion and stereotypy in C57BL/6 mice. *Eur. J. Pharmacol.* 580 161–168. 10.1016/j.ejphar.2007.07.071 18053981 PMC2705961

[B35] Rolls E. T. (2019). The cingulate cortex and limbic systems for action, emotion, and memory. *Handb. Clin. Neurol.* 166 23–37. 10.1016/B978-0-444-64196-0.00002-9 31731913

[B36] Rudebeck P. H. Buckley M. J. Walton M. E. Rushworth M. F. (2006). A role for the macaque anterior cingulate gyrus in social valuation. *Science* 313 1310–1312. 10.1126/science.1128197 16946075

[B37] Sawahata M. Asano H. Nagai T. Ito N. Kohno T. Nabeshima T. (2021). Microinjection of Reelin into the mPFC prevents MK-801-induced recognition memory impairment in mice. *Pharmacol. Res.* 173:105832. 10.1016/j.phrs.2021.105832 34450306

[B38] Shao D. Cao Z. Fu Y. Yang H. Gao P. Zheng P. (2021). Projection from the basolateral amygdala to the anterior cingulate cortex facilitates the consolidation of long-term withdrawal memory. *Addict. Biol.* 26:e13048. 10.1111/adb.13048 33973711

[B39] Tamamaki N. Yanagawa Y. Tomioka R. Miyazaki J. Obata K. Kaneko T. (2003). Green fluorescent protein expression and colocalization with calretinin, parvalbumin, and somatostatin in the GAD67-GFP knock-in mouse. *J. Comp. Neurol.* 467 60–79. 10.1002/cne.10905 14574680

[B40] Tandon R. Gaebel W. Barch D. M. Bustillo J. Gur R. E. Heckers S. (2013). Definition and description of schizophrenia in the DSM-5. *Schizophr. Res.* 150 3–10. 10.1016/j.schres.2013.05.028 23800613

[B41] Thomases D. R. Cass D. K. Tseng K. Y. (2013). Periadolescent exposure to the NMDA receptor antagonist MK-801 impairs the functional maturation of local GABAergic circuits in the adult prefrontal cortex. *J. Neurosci.* 33 26–34. 10.1523/JNEUROSCI.4147-12.2013 23283319 PMC3544161

[B42] Ungvari Z. Tarantini S. Hertelendy P. Valcarcel-Ares M. N. Fulop G. A. Logan S. (2017). Cerebromicrovascular dysfunction predicts cognitive decline and gait abnormalities in a mouse model of whole brain irradiation-induced accelerated brain senescence. *Geroscience* 39 33–42. 10.1007/s11357-017-9964-z 28299642 PMC5352588

[B43] van Os J. Kapur S. (2009). Schizophrenia. *Lancet* 374 635–645. 10.1016/S0140-6736(09)60995-8 19700006

[B44] Winship I. R. Dursun S. M. Baker G. B. Balista P. A. Kandratavicius L. Maia-de-Oliveira J. P. (2019). An overview of animal models related to Schizophrenia. *Can. J. Psychiatry* 64 5–17. 10.1177/0706743718773728 29742910 PMC6364139

[B45] Woo T. U. Walsh J. P. Benes F. M. (2004). Density of glutamic acid decarboxylase 67 messenger RNA-containing neurons that express the N-methyl-D-aspartate receptor subunit NR2A in the anterior cingulate cortex in schizophrenia and bipolar disorder. *Arch. Gen. Psychiatry* 61 649–657. 10.1001/archpsyc.61.7.649 15237077

[B46] Wu J. Zou H. Strong J. A. Yu J. Zhou X. Xie Q. (2005). Bimodal effects of MK-801 on locomotion and stereotypy in C57BL/6 mice. *Psychopharmacology (Berl)* 177 256–263. 10.1007/s00213-004-1944-1 15290006

[B47] Yang Y. Wang J. Z. (2017). From structure to behavior in basolateral amygdala-hippocampus circuits. *Front. Neural. Circuits* 11:86. 10.3389/fncir.2017.00086 29163066 PMC5671506

[B48] Young J. W. Powell S. B. Risbrough V. Marston H. M. Geyer M. A. (2009). Using the MATRICS to guide development of a preclinical cognitive test battery for research in schizophrenia. *Pharmacol. Ther.* 122, 150–202. 10.1016/j.pharmthera.2009.02.004 19269307 PMC2688712

[B49] Zeeb F. D. Winstanley C. A. (2011). Lesions of the basolateral amygdala and orbitofrontal cortex differentially affect acquisition and performance of a rodent gambling task. *J. Neurosci.* 31 2197–2204. 10.1523/JNEUROSCI.5597-10.2011 21307256 PMC6633057

