# Supplementary material for: Activation of basolateral amygdala to anterior cingulate cortex circuit alleviates MK-801 induced social and cognitive deficits of schizophrenia
Source: Front Cell Neurosci. 2022 Dec 22;16:1070015. doi: 10.3389/fncel.2022.1070015 (PMC9813383; doi:10.3389/fncel.2022.1070015)
Supplement: Supplementary file 1 [file Data_Sheet_1.docx]

Supplementary Material

# Supplementary Figures and Tables

## Supplementary Figures

**
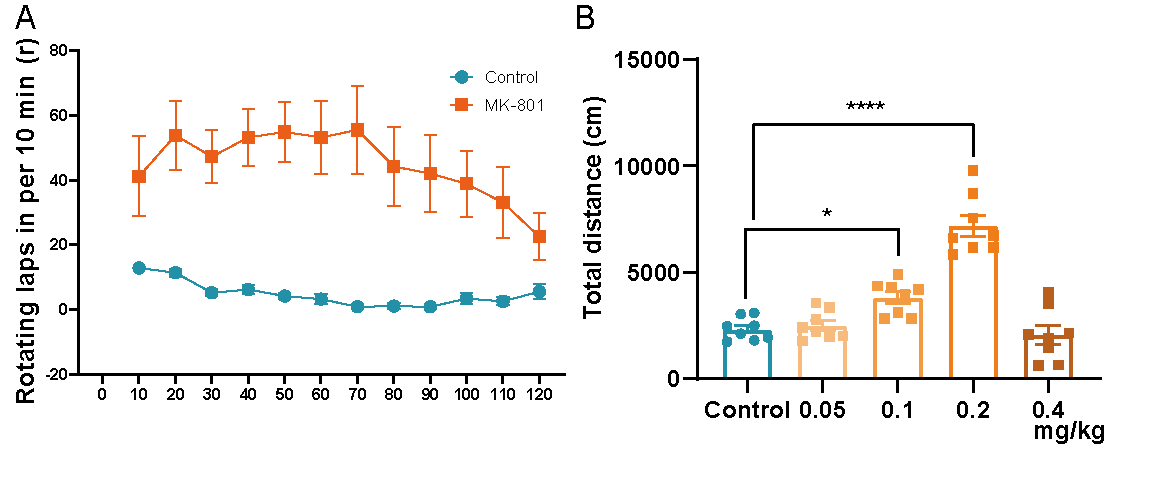
**

**Supplementary Figure 1. Positive behaviors of mice after the injection of different concentrations of MK-801.**

(A) Rotating laps in every 10 minutes after MK-801 was injected at a dose of 0.2 mg/kg (Control n=6 mice, MK-801 n=6 mice).

(B) Total distance that mice traveled after injection of different concentrations of MK-801 (One-factor ANOVA, Control n=8 mice, MK-801 0.05 mg/kg n=8 mice, MK-801 0.1 mg/kg n=8 mice, MK-801 0.2 mg/kg n=8 mice, MK-801 0.4 mg/kg n=8 mice). Data are shown as the means ± SEM. * indicates a significant difference between groups. **p* < 0.05 and *****p* < 0.0001.


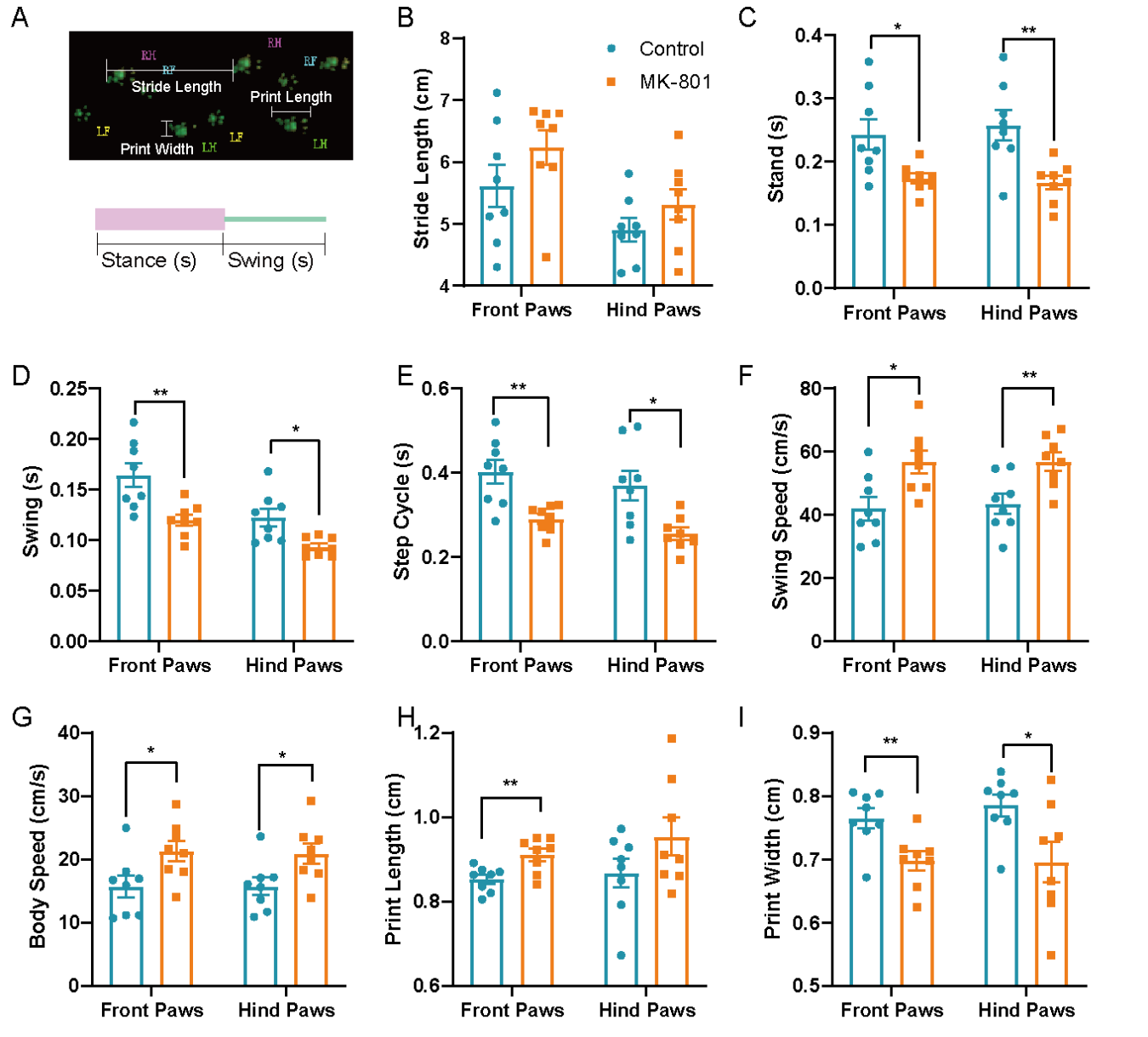


**Supplementary Figure 2. MK-801 caused gait disorders in mice.**

(A) Diagram of the CatWalk test and the main indicators of the analysis.

(B) The stride length did not change after the MK-801 injection (n=8 mice in each group).

(C) The stand time was significantly decreased after the MK-801 injection compared with control mice.

(D) The swing time was significantly decreased after the MK-801 injection compared with control mice.

(E) The step cycle was significantly decreased after the MK-801 injection compared with control mice.

(F) The swing speed was significantly increased after the MK-801 injection compared with that of control mice.

(G) The body speed was significantly increased after the MK-801 injection compared with that of control mice.

(H) The print length of the front paws was significantly increased after the MK-801 injection compared with control mice, while no change was observed in the print length of the hind paws.

(I) The print width was significantly increased after the MK-801 injection compared with control mice. Data are shown as the means ± SEM. * indicates a significant difference between groups. **p* < 0.05 and ***p* < 0.01.


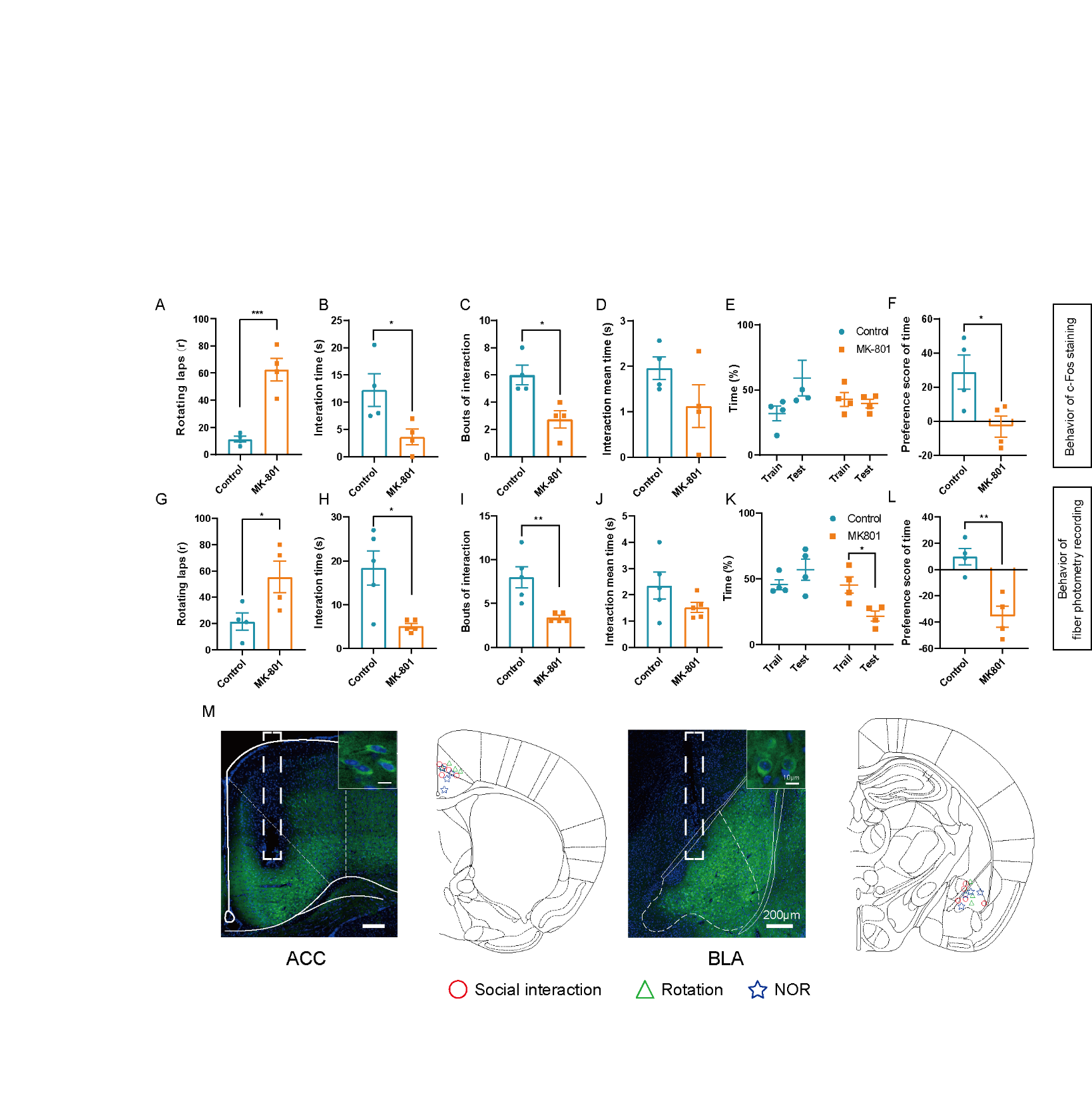


**Supplementary Figure 3.** Results of behavioral tests for c-Fos staining and in vivo fiber photometry recordings.

(A) The rotating laps of MK-801-injected mice were significant increase compared with control mice (unpaired t-test, n=4 mice in each group) before c-Fos staining.

(B-D) The results of social interaction test before c-Fos staining: The interaction time (B, unpaired t-test, n=4 mice in each group) and bouts of the interaction (C, unpaired t-test, n=4 mice in each group) were significant decrease compared with control mice before c-Fos staining. While the interaction mean time of MK-801-injected had a decreasing trend without statistical difference (D, unpaired t-test, n=4 mice in each group).

(E-F) The time spent in exploring the novel object (E, paired t-test, n=4 mice in each group) and the preference score of time (F, unpaired t-test, n=4 mice in each group) of the MK-801-injected mice were significant decrease compared with control mice before c-Fos staining.

(G) The rotating laps of MK-801-injected mice and control mice (unpaired t-test, n=4 mice in each group) during fiber photometry recording.

(H-J) The results of social interaction test during fiber photometry recording: The interaction time (H, Two-tailed unpaired separate variance estimation t-test, n=5 mice in each group), bouts of the interaction (I, Mann-Whitney U test, n=5 mice in each group) and interaction mean time of MK-801-injected mice and control mice (J, unpaired t-test, n=5 mice in each group).

(K-L) The time spent exploring the novel object (K, Wilcoxon signed-rank test/paired t-test, n=4 mice in each group) and the preference score of time (L, unpaired t-test, n=4 mice in each group) of the MK-801-injected mice and control mice during fiber photometry recording.

(M) Representative confocal images showing the optical implanted areas of the ACC and BLA for fiber photometry recording.


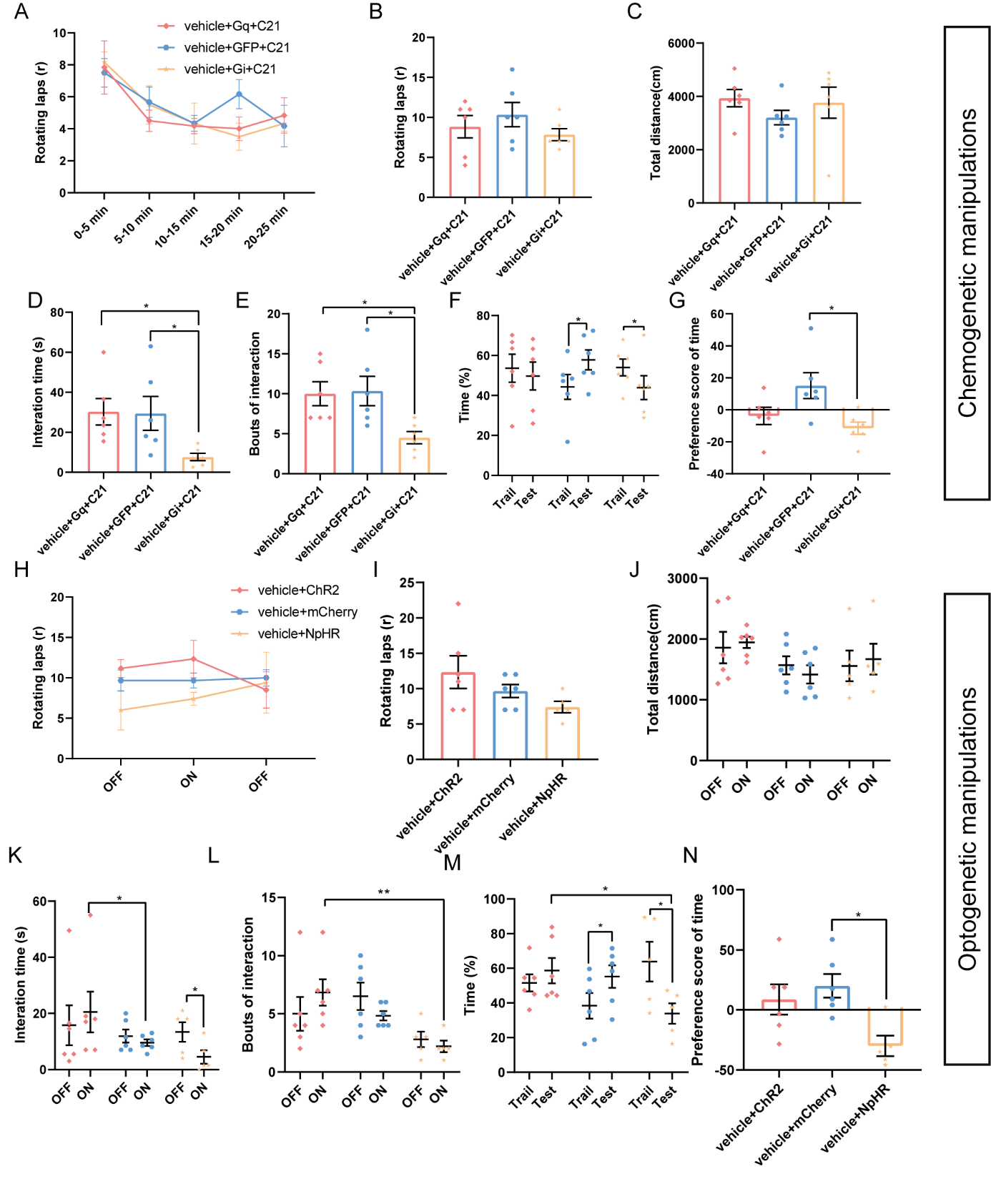


**Supplementary Figure 4. Chemogenetic and optogenetic regulation of BLA-ACC projecting neurons in vehicle injected mice.**

(A-C) The results of the rotational behavior test (A, Friedman's M test; B, One-factor ANOVA, vehicle+Gq+C21 n=6 mice, vehicle+GFP+C21 n=6 mice, vehicle+Gi+C21 n=6 mice) and OFT (C, One-factor ANOVA, vehicle+Gq+C21 n=6 mice, vehicle+GFP+C21 n=6 mice, vehicle+Gi+C21 n=6 mice) after chemogenetic regulation of BLA-ACC circuit in vehicle injected mice.

(D-E) The results of the social interaction test (One-factor ANOVA, vehicle+Gq+C21 n=6 mice, vehicle+GFP+C21 n=6 mice, vehicle+Gi+C21 n=6 mice) after chemogenetic regulation of BLA-ACC circuit in vehicle injected mice.

(F-G) The results of the percentage of time that mice spent in exploring the novel object (F, One-factor ANOVA) and the preference scores of NOR test after chemogenetic regulation of BLA-ACC circuit (G, One-factor ANOVA, vehicle+Gq+C21 n=6 mice, vehicle+GFP+C21 n=6 mice, vehicle+Gi+C21 n=6 mice).

(H-J) The results of the rotational behavior test (H, Friedman's M test; I, One-factor ANOVA, vehicle+ChR2 n=6 mice, vehicle+mCherry n=6 mice, vehicle+NpHR n=5 mice) and OFT (J, One-factor ANOVA, vehicle+ChR2 n=6 mice, vehicle+mCherry n=6 mice, vehicle+NpHR n=5 mice) after optogenetic regulation of BLA-ACC circuit in vehicle injected mice.

(K-L) The results of the social interaction test ( One-factor ANOVA/ Kruskal-Wallis H test, vehicle+ChR2 n=6 mice, vehicle+mCherry n=6 mice, vehicle+NpHR n=5 mice) after optogenetic regulation of BLA-ACC circuit in vehicle injected mice.

(M-N) The results of the percentage of time that mice spent in exploring the novel object (M, One-factor ANOVA) and the preference scores of NOR test after optogenetic regulation of BLA-ACC circuit (N, One-factor ANOVA, vehicle+ChR2 n=6 mice, vehicle+mCherry n=6 mice, vehicle+NpHR n=5 mice). Data are shown as the means ± SEM. * indicates a significant difference between groups. **p* < 0.05, ***p* < 0.01, ****p* < 0.001, and *****p* < 0.0001.


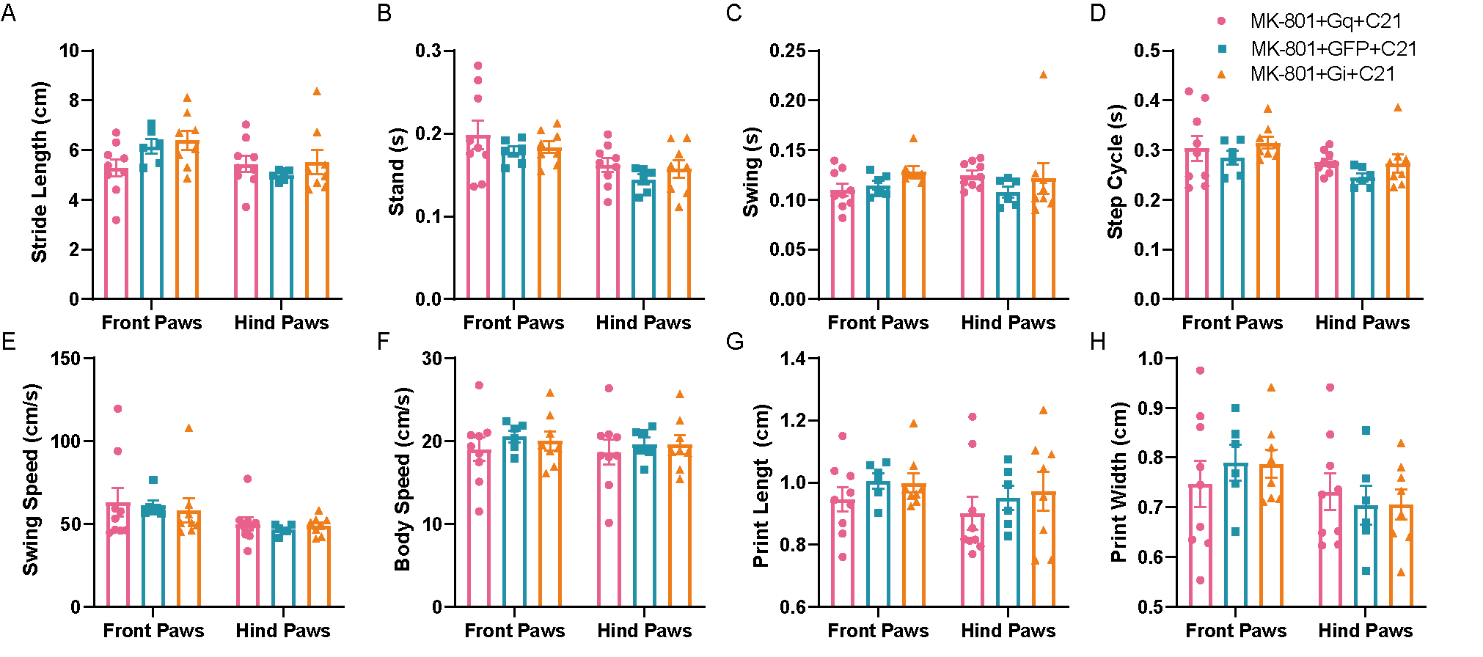


**Supplementary Figure 5. Chemogenetic regulation of BLA-ACC projecting neurons had no effect on the gait of mice after the MK-801 injection.**

(A-H) No effect on the gait of mice was observed after the MK-801 treatment along with C21 injection (Kruskal-Wallis H test/ One-factor ANOVA, Gq n=9 mice, GFP n=6 mice, Gi n=8 mice).


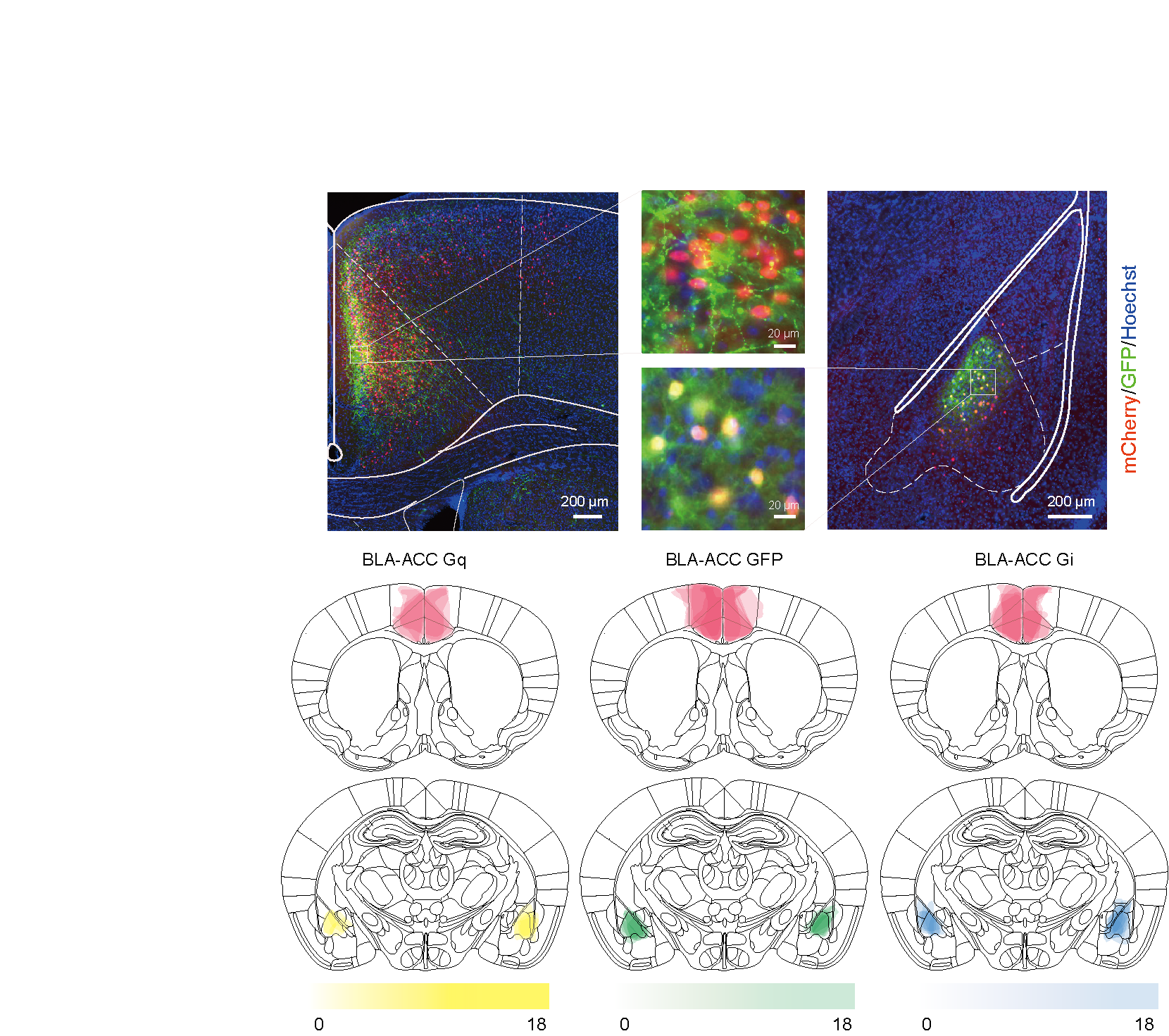


**Supplementary Figure 6. Verification of the injection site in the BLA and ACC of C57BL/6J mice for the chemogenetic regulation.**


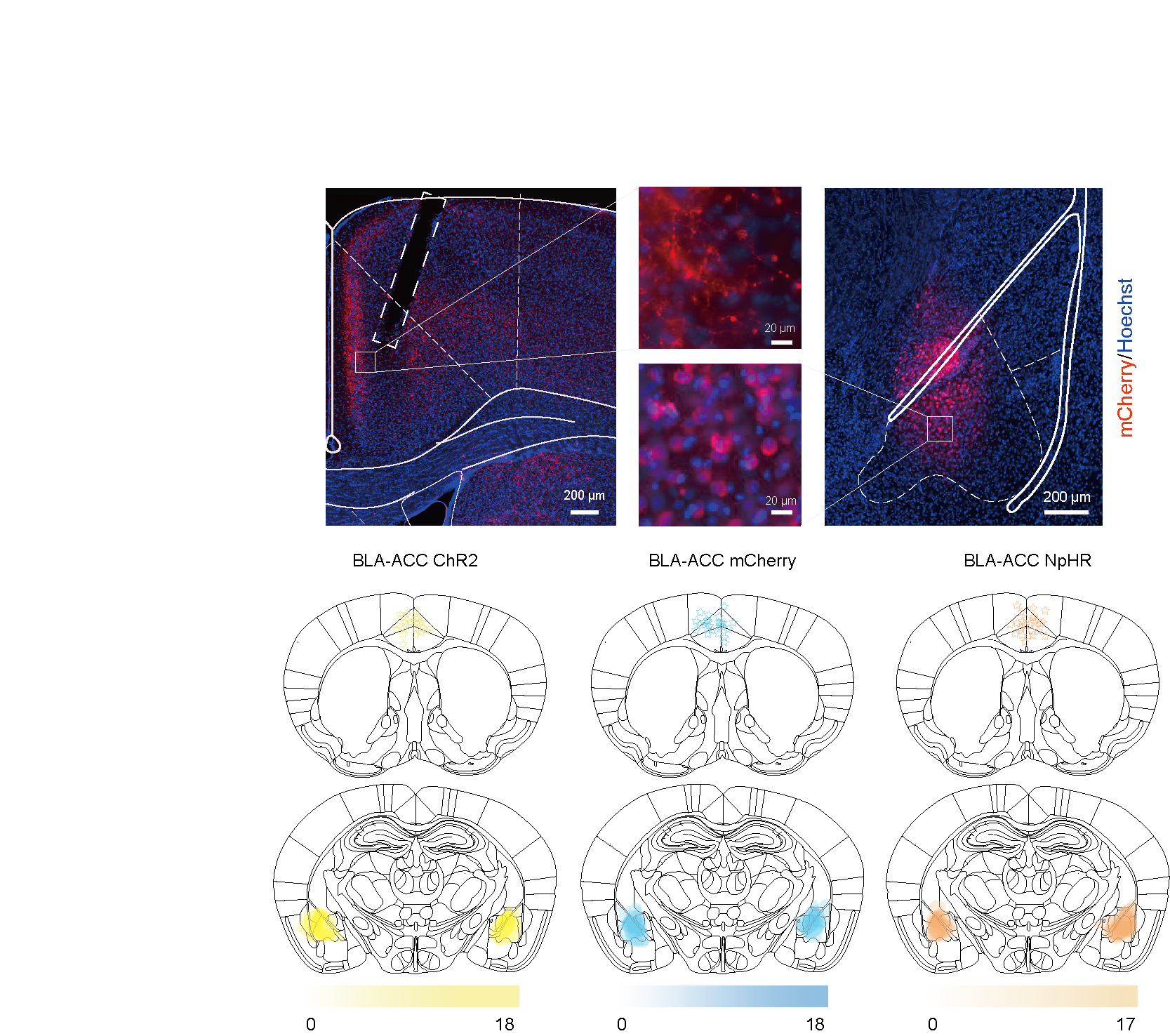


**Supplementary Figure 7. Verification of the injection site in the BLA and fiber implantation in the ACC for optogenetic modulation of C57BL/6J mice.**


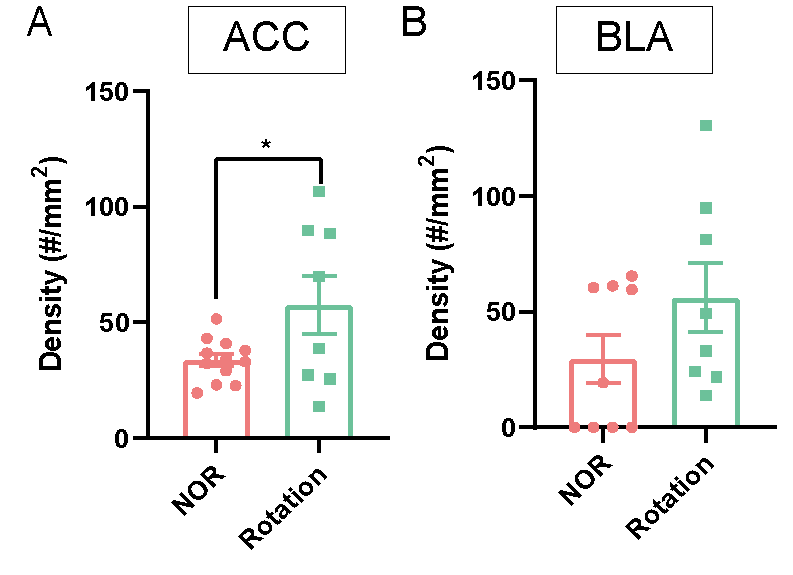


**Supplementary Figure 8. Comparison of c-Fos-positive cells in rotational behavior and NOR test.**

(A-B) The c-Fos-positive cells in the ACC (A, unpaired t-test, Rotation n=8 slices/4 mice, NOR n=12 slices/4 mice) and BLA (B, Mann-Whitney U test, Rotation n=8 slices/4 mice, NOR n=9 slices/4 mice) of the mice in rotational behavior and NOR test.
